# Transcriptomic and proteomic analysis of *Hemidactylus frenatus* during initial stages of tail regeneration

**DOI:** 10.1038/s41598-021-83283-0

**Published:** 2021-02-11

**Authors:** Sai Pawan Nagumantri, Sarena Banu, Mohammed M. Idris

**Affiliations:** CSIR-CCMB, Uppal Road, Habsiguda, Hyderabad, 500007 India

**Keywords:** Developmental biology, Proteomic analysis

## Abstract

Epimorphic regeneration of appendages is a complex and complete phenomenon found in selected animals. *Hemidactylus frenatus*, house gecko has the remarkable ability to regenerate the tail tissue upon autotomy involving epimorphic regeneration mechanism. This study has identified and evaluated the molecular changes at gene and protein level during the initial stages, i.e., during the wound healing and repair mechanism initiation stage of tail regeneration. Based on next generation transcriptomics and De novo analysis the transcriptome library of the gecko tail tissue was generated. A total of 254 genes and 128 proteins were found to be associated with the regeneration of gecko tail tissue upon amputation at 1, 2 and 5-day post amputation (dpa) against control, 0-dpa through differential transcriptomic and proteomic analysis. To authenticate the expression analysis, 50 genes were further validated involving RTPCR. 327 genes/proteins identified and mapped from the study showed association for Protein kinase A signaling, Telomerase BAG2 signaling, paxillin signaling, VEGF signaling network pathways based on network pathway analysis. This study empanelled list of transcriptome, proteome and the list of genes/proteins associated with the tail regeneration.

## Introduction

Regeneration is a sequential process specifically controlled by cellular mechanisms to repair or replace tissue or organ from an injury. The mass of undifferentiated cells (blastema) surrounding the injured tissue results in the formation of fully functional replica or imperfect replica which enacts the phenomenon of epimorphic regeneration^1,2^. The ability to regenerate a full limb is absent in mammals but urodeles; teleost and anamniotes have the propensity to replace limbs, spinal cords, nervous system, heart, tail and, other body parts^1–3^. To understand the mechanistic framework of regeneration, research studies have been majorly executed in urodeles amphibians^4^ and teleost fishes^1^. Though amniotes i.e. lizards are closely related to mammals and other vertebrates, limited studies^5,6^ have been carried towards understanding the molecular mechanism of regeneration.

Lizard capacity to self-detach or amputate their tail in flight response from predators is known as the shedding of the tail or caudal autotomy^7^. These stages include exudation of excessive blood loss; minimizing muscle and bone tissue damage; controlled vascularisation; promotes wound epithelium and further unsegmented remodelling^8^. The detachment of tail activates multiple cellular responses which spurts the blastema mediated cell proliferation^9^, angiogenesis, remodelling and generating a replacement. Studies related to the patterning of successful regenerated autotomized tail or replica consists mainly of nerve cells networking, unsegmented cartilaginous, muscle cells, blood vessels and differential remodelling of cells which make this an interesting research to comprehend the cellular and molecular mechanisms associated with the developmentof regenerated tail^10–12^. With the elucidation of the genes and their associated proteins involved in regeneration, there can be a possibility of understanding why in human’s regeneration is restricted as compared to anamniotes. This also might pave in giving insights into spinal injuries and the fabrication of replacement therapies^13^. Research on the regeneration of tail and its governing molecular mechanism in other lizard species like *Podarcis muralis*^14,15^; *Hemidactylus flaviviridis*^16^; Green Anole^17,18^; and also in other vertebrates like axolotl^19^ have been carried out related to histology, proteomics and genomics^20,21^. Though many genes or proteins have been known still the underlying mechanisms of these molecules remains ambiguous. In this study for the first time we have used common house Gecko, *Hemidactylus frenatus*^22^ as a model animal to study the genes/proteins associated with tail regeneration particularly during the initial stages of tail regeneration. *Hemidactylus frenatus* have been studied generally for their behavioural, habitat diversification and regenerative abilities^9,23–26^.

Lately, molecular and gene-specific studies focus on the role of genes like SOX9, PAX7, BMP, FGF, Wnt, MMP’s which are usually associated with regeneration^20,27–31^. The expression of SOX9 during cartilage formation, PAX7 associating in tissue morphogenesis^28, 31^, BMP6 and other protein expressions were known^29,30^. Constitutive expression and regulation of these genes have revealed a significant role during the developmental stages of regeneration. The slight alteration in any of the crucial genes regulation might results in a disrupted replacement^32^. Thus, the activation and intricate cross-talk of these molecules during the process are still unaddressed and need to be better understood.

In this research, we sought to study the molecular mechanisms of lost tail regeneration process during wound healing and initiation of repair mechanism stage (0-dpa, 1-dpa, 2-dpa, and 5-dpa). The 1–5-dpa stage would shed light on involvement of genes or proteins during wound healing and initiation of proliferation/activation of multiple cells initiating blastema formation which eventually leads to the regeneration of tail. The initial stages (i.e. wound healing) are responsible for wound epithelium and initiating apical epidermal cap (AEC) in amphibians which regulates blastema formation^4^ whereas in lizards- similar to AEC an uncertain apical epidermal PEG(AEP)^33^ forms. Though, during the initial stages, the activity of the molecules regulating the regeneration process is not known which is crucial to instigate the replacement of the lost tissue. The study of prelude stages of regeneration has been carried out in zebrafish caudal fin^34^; other species^35^ and shown changes in the regulation of genes or proteins. However, this stage-specific study has not been carried out in this species- and it is necessary for deciphering the crucial molecules which kick starts the regenerative mechanism in these amniotes. Here, we list out the involvement of crucial genes or proteins and their differential expression associated with the molecular processes in tail regeneration through transcriptome and proteome approaches.

## Results

### Transcriptomic analysis of regeneration

A total of 42,551 transcripts sequences were obtained from the NGS analysis of all four tail tissue samples. All the sequences were obtained from a total of ~ 30 million reads from each sample. De novo and functional annotation yielded 39,953 transcripts with highest similarity with *Gekko japonicas* (41.3%) and no blast hit (49%) to any reptilian database. The transcripts were submitted to NCBI and obtained accession number (SUB6743288 & PRJNA587566; https://dataview.ncbi.nlm.nih.gov/object/PRJNA587566). The transcripts were further translated to obtain the protein sequence database for proteomic analysis.

**Figure 1:**
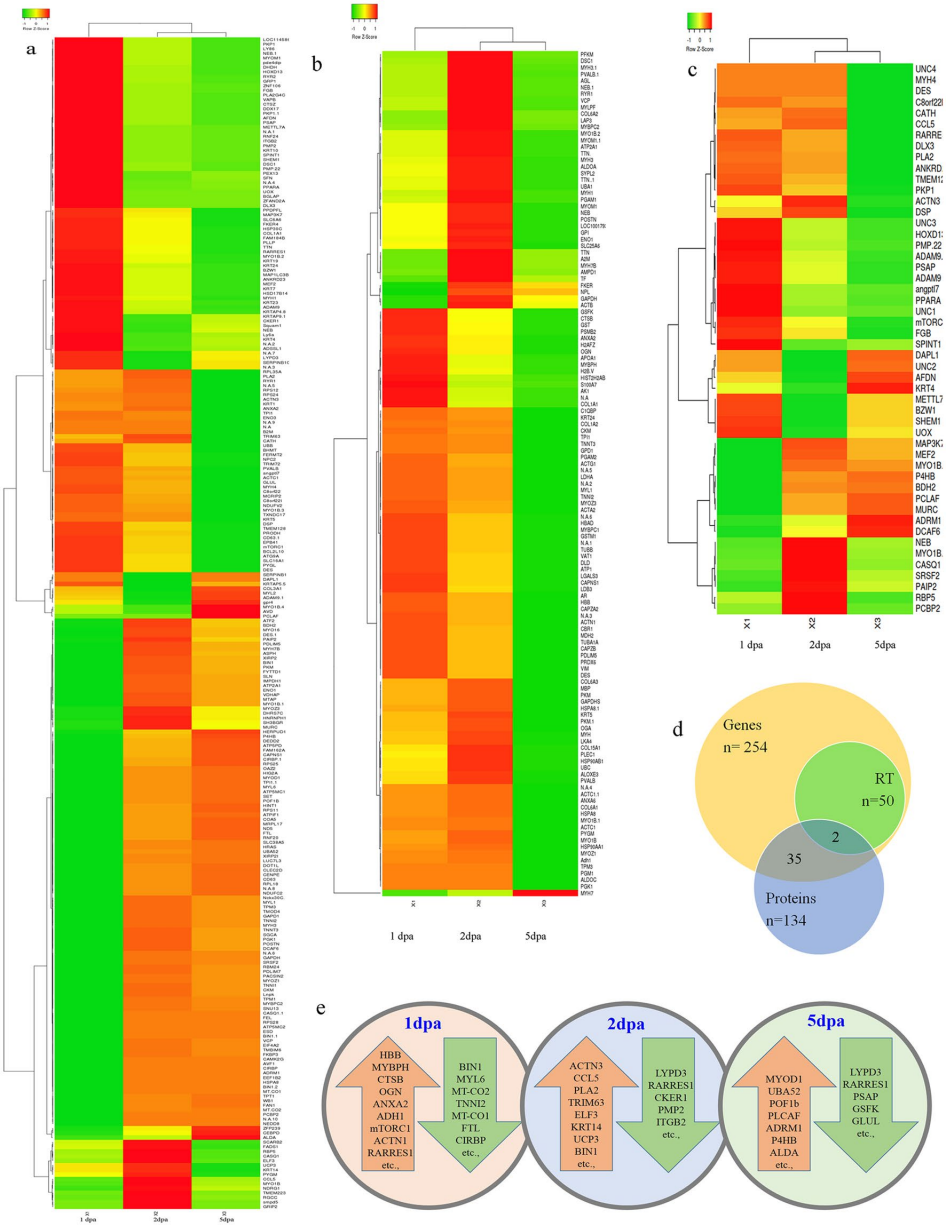
(**a**) Heap map expression of *Hemidactylus frenatus* transcript during tail tissue regeneration. (**b**) Heat map expression of differentially expressed proteins which are associated with tail regeneration; (**c**) Heat map expression of various genes differentially regulated during regeneration based on RTPCR analysis. (**d**) Venn diagram for number genes identified from Transcriptomics, proteomics and Real time PCR based gene expression analysis; e. Batches of genes/proteins which are differentially expressed at 1, 2 and 5-dpa. Genes/proteins which are in the red/organge arrow indicates the upregulation and in the green arrow indicates the downregulation.

A total of 417 genes were found to be associated with regeneration of gecko tail tissue for having at least one log fold change in one of its regenerating time points significantly. 254 genes were selected for further analysis such as heat map and network pathway analysis for having their expression pattern in all their regenerating time points (Fig. 1a; Supplementary Table S1). 26, 38 and 29% of identified genes were found to be upregulated for having > 1 log fold change in their expression during the regeneration process at 1, 2 and 5-dpa. Similarly, 54, 35 and 46% of genes were found to be downregulated by > − 1 log fold during regeneration at their respective 1, 2 and 5-dpa time points.

Gene Ontology (GO) analysis of differential expressed genes (DEG’s) were carried out against Anole Lizard database to determine the biological process. The DEG’s GO of all the 254 genes revealed highly enriched functional roles and almost 80 enriched terms were listed including genes responsible for muscle system process, muscle tissue morphogenesis, cellular component organization and biogenesis, immune system process and regulation (Supplementary Table S2). In GO of upregulated genes at 2-dpa & 5-dpa, we identified only 5 & 22 high enriched terms whereas at 1-dpa no significant term was identified. Downregulated genes at 1-dpa, 2-dpa & 5-dpa found to be associated with 84, 24 & 5 high enriched terms respectively. The GO terms in upregulated genes included regulation in response to stimulus, anatomical structure morphogenesis, muscle system process, mitochondrial protein complex, cytoskeletal protein binding and among others.

We also observed a significant fivefold change in some of the genes associated with developmental process; anatomical structural development/morphogenesis and molecular functions. Erythrocyte membrane protein (EPB41), retinoic acid receptor responder protein 1 (RARRES1), alpha-actinin-3 (ACTN3), ETS-related transcription factor (Elf-3), fatty acid desaturase 1-like (FADS1), Prolyl 4-hydroxylase beta polypeptide (P4HB), NADH dehydrogenase subunit 5 (NDUFV2), Pancreatic progenitor cell differentiation and proliferation factor (PPDPFL), PCNA-associated factor(PCLAF) and few uncharacterized genes were found to be upregulated for having more than 5 log fold upregulation of gene expression. Similarly, RYR1, MYO1B, MYL2, MYL6, CD63, MYLPF, PCLAF, MYO16, GRIP2, BIN1, LUC7L3, FGB, ANXA2, MYOZ3 and among other genes were found to be regulated austerely in all time points (Supplementary Table S1). Most of the genes involved were associated with supramolecular assembly, muscle cells regulation/contraction and multiprotein signalling complex. This in turn relates to crucial involvement and regulation of genes in initiating process for haemostasis & wound healing phase.

Cluster hierarchical heat map analysis of DEGs revealed group of genes which were undergoing upregulation at 1-dpa where found to be downregulated at 2 and 5-dpa and vice versa with downregulated genes at 1-dpa (Fig. 1a). Genes with crucial biological functions were identified and found involved in the onset of blastemal formation or AEP during wound healing phase. Some major genes associated with growth, cellular proliferation & biogenesis, immune response activation, multicellular organismal process, ion binding, transporter activity, cellular response to stimulus were identified on reference with Anole Lizard database are MYOD1, TNNI1, ASPH, TPT1, CD63, BIN1, NDRG1, CEBPD, POF1B, POSTN, TMBIM6, RPS25, CIBP, HERPUD1, UBA52, HINT1, FAN1, FAM162A, RNF20, LUC7L3 and SLC38A5. In this study, we observed mostly muscle contraction, signalling, structural molecule activity, anatomical morphogenesis and developmental genes. The analysis further reveals that 1-dpa expression is out rooted with clustered 2 and 5-dpa based on cluster analysis for the three regenerating time points against the control tissue expression.

### Proteomic analysis of regeneration

A total of 128 proteins were found to be differentially regulated during the early stage of regeneration of gecko tail tissues for having a minimum of one log fold changes significantly (Supplementary Table S3). 36 and 33% of proteins were found to be upregulated for having > 1 log fold change at 1 and 2-dpa with less than 10% proteins to be downregulated in the same points. Whereas 67% of proteins were found to be downregulated for the regeneration mechanism at 5-dpa with only one protein upregulated in the same time point. Haemoglobin subunit-β (HBB)^29^, Myosin Binding Protein-H (MYBPH)^30^, Annexin A2, Cathepsin B, Mimecan (OGN), galectin (LGALS3) were few of the proteins upregulated at 1-dpa and subsequently downregulated at 2 and 5-dpa. Polyubiquitin-C (UBC), MYLPF, PVALB, LAP3 and MYH are few of the proteins upregulated at 2-dpa and MYH7 found to be upregulated at 5-dpa. A total of 37 proteins which were differentially regulated at the proteome level were also found to be differentially regulated at gene level based on the transcriptomics analysis (Fig. 1b,d). Like transcriptome expression pattern, proteome expressions were also found to be associated with regeneration by clustering of 2 and 5-dpa (Fig. 1c). DES and NEB were the 2 genes/proteins which was identified from the transcriptomics and proteomic analysis were validated involving RTPCR analysis (Fig. 1d). All the proteins which were upregulated at 1-dpa were found to be downregulated either at 2 or 5-dpa. Similarly, all the 1-dpa downregulated proteins were found to be upregulated at 2 or 5-dpa (Fig. 1e).

### Upregulation and downregulation of genes/proteins

Based on the combined transcriptomic and proteomic analysis it was observed that 29, 36 and 20% of genes/proteins were found to be upregulated by more than one log-fold for the initiation of regeneration mechanism in gecko. Similarly, 39, 24 and 53% of genes/proteins were found to be downregulated for the regeneration mechanism. Crucial genes/proteins such as Haemoglobin subunit-β (HBB)^31^, Myosin binding proteins-H (MYBPH)^32^, AnnexinA2 (ANXA2), CTSB, OGN, ANXA2, ADH1, mTORC1, ACTN1 and RARRES1 were found to be upregulated and associated with pain and inflammation, DNA repair, ubiquitination, proteasomal activities and cellular morphology at 1-dpa (Fig. 1e). Subsequent downregulation of these genes/proteins were observed on 2-dpa indicating the downregulation of inflammation and PTMs. Whereas cellular and other skeletal & muscle development process were found to be upregulated on 2-dpa (Fig. 1e) through the upregulation of ACTN3, CCL5, PLA2, TRIM63, ELF3, UCP3 and BIN1. Similarly, on 5-dpa all the genes/proteins involved in inflammation and various PTMs were found to be downregulated which were found upregulated at 1 and 2-dpa (Fig. 1e).

### Validation of gene expression

**Figure 2:**
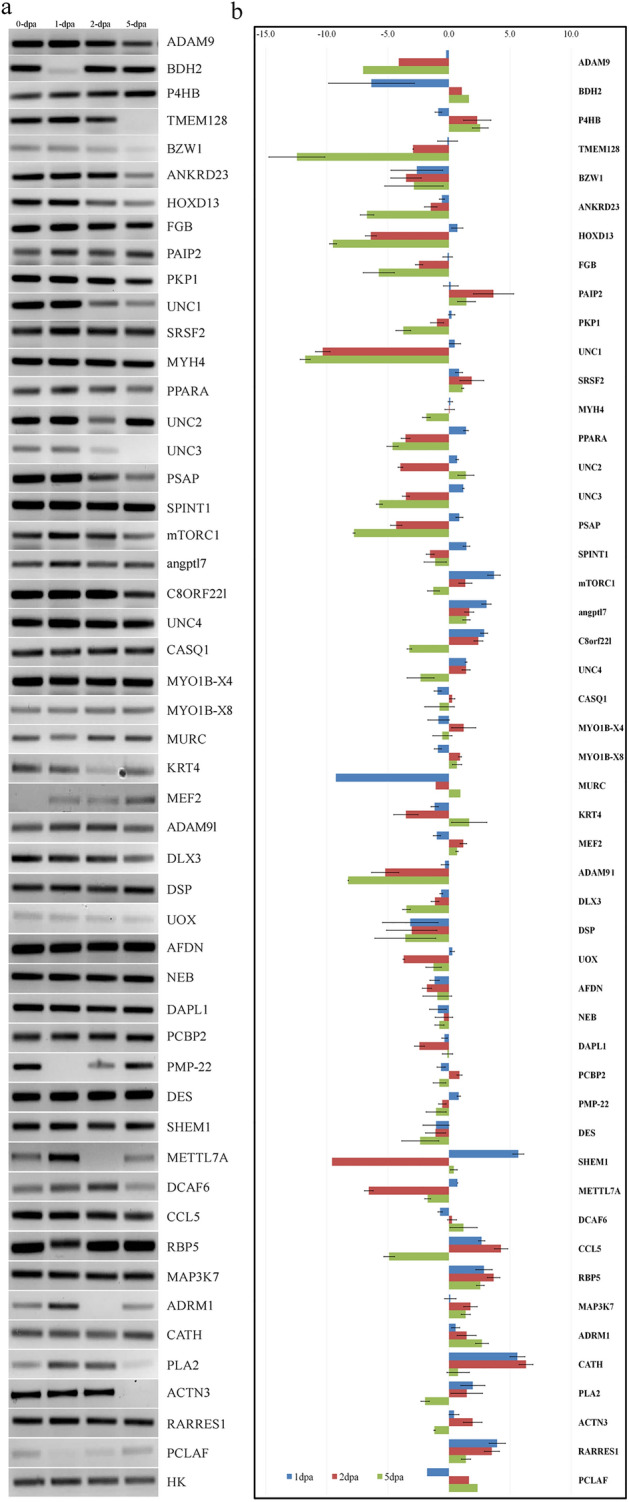
RTPCR analysis of differentially expressed transcripts. (**a**) RTPCR Gel image of all the 50 genes along with housekeeping gene for 0-dpa (control), 1-dpa, 2-dpa and 5-dpa. (**b**) Bar diagram of expression visualising the up and downregulation of the 50 genes.

RTPCR analysis of 50 genes selected for the validation study showed significant differential expression of the genes for the regeneration mechanism (Fig. 2a,b). Almost all the up and downregulated genes which were identified from the transcriptomic analysis were found to be associated with regeneration through differential regulation based on RTPCR analysis (Fig. 1c). Heat map analysis of the gene expression based on RTPCR analysis also showed association of 2 and 5-dpa as cluster against 1-dpa expression.

### Network pathway analysis

A total of 327 genes/proteins were found mapped by the Ingenuity Pathway analysis software for various canonical network and pathways of human and mouse database. The major molecular and cellular functions associated with the differentially regulated genes/proteins during regeneration are cellular assembly & organization, cellular compromise, transcriptional & translational regulatory genes/proteins, DNA binding protein, cellular functions & maintenance and Cellular development along with structural constituent of cytoskeleton proteins. The major physiological system development and functions associated with regeneration mechanism are skeletal & muscular system development, embryonic development, organ development and tissue development.

**Figure 3:**
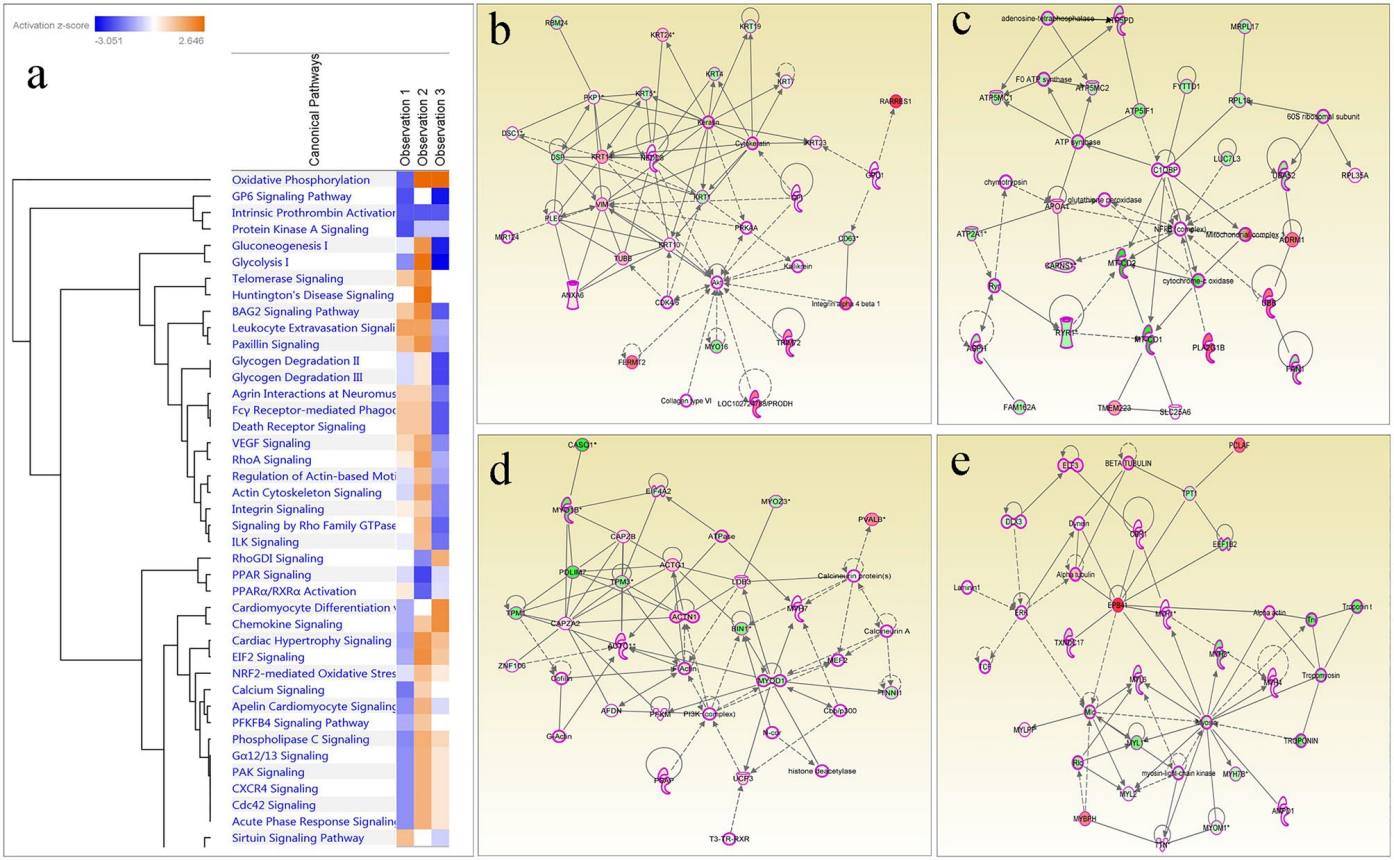
(**a**) Canonical Pathway associated with the differentially expressed genes/proteins for tail regeneration; (**b**) Cell morphology and Embryonic development network pathway; (**c**) Cellular assembly and organization network pathway; (**d**) Organ and organismal development network pathway; (**e**) Skeletal and Muscular development network pathway.

The most significant canonical pathways which were found to be associated with genes/proteins data obtained from transcriptomics and proteomics analysis were GP6 signaling pathway, Protein kinase A signaling, Telomerase signaling BAG2 signaling, paxillin signaling, VEGF signaling and various metabolic pathways (Fig. 3a). The major network pathways associated with the identified and dysregulated genes/proteins based on differential analysis includes Cell morphology & Embryonic development, Cellular assembly & organization, Organ & organismal development and Skeletal & Muscular development network pathways.

Cell morphology and Embryonic development network pathway (Fig. 3b) was found to be associated with Akt, ANXA6, CD63, CDK4/6, Collagen type VI, Cytokeratin, DSC1, DSP, FERMT2, GPD1, GPI, Integrin alpha 4 beta 1, Kallikrein, Keratin, KRT1, KRT10, KRT14, KRT19, KRT23, KRT24, KRT4, KRT5, KRT7, LOC102724788/PRODH, MIR124, MYO16, NEDD8, PKP1, PLEC, PRKAA, RARRES1, RBM24, TRIM72, TUBB, VIM genes/proteins which were found differently expressed during the regeneration of tail tissue. Similarly Cellular assembly and organization network pathway (Fig. 3c) was found associated with 60S ribosomal subunit, adenosine-tetraphosphatase, ADRM1, APOA1, ASPH, ATP synthase, ATP2A1, ATP5IF1, ATP5MC1, ATP5MC2, ATP5PD, C1QBP, CAPNS1, chymotrypsin, cytochrome-c oxidase, F0 ATP synthase, FAM162A, FAN1, FYTTD1, glutathione peroxidase, LUC7L3, Mitochondrial complex 1, MRPL17, MT-CO1, MT-CO2, NFkB (complex), PLA2G1B, RPL18, RPL35A, Ryr, RYR1, SLC25A6, TMEM223, UBA52, UBB genes/proteins. Genes/proteins associated with cellular assembly and organization were also associated with mitochondrial protein synthesis, protein transport, signal transduction, and cytoskeleton remodelling.

Organ and organismal development network pathway (Fig. 3d) was found associated with ACTC1, ACTG1, ACTN1, AFDN, ATPase, BIN1, Calcineurin A, Calcineurin protein(s), CAPZA2, CAPZB, CASQ1, Cbp/p300, Cofilin, EIF4A2, F Actin, G-Actin, histone deacetylase, LDB3, MEF2, MYH7, MYO1B, MYOD1, MYOZ3, N-cor, PDLIM7, PFKM, PI3K (complex), PSAP, PVALB, T3-TR-RXR, TNNI1, TPM1, TPM3, UCP3 and ZNF106 genes/prtoeins. Skeletal and Muscular development network pathway (Fig. 3e) was fond associated with Alpha actin, Alpha tubulin, AMPD1, BETA TUBULIN, CBR1, DLX3, Dynein, EEF1B2, ELF3, EPB41, ERK, Laminin1, Mlc, MYBPH, MYH1, MYH3, MYH4, MYH7B, MYL1, MYL2, MYL6, MYLPF, MYOM1, Myosin, myosin-light-chain kinase, PCLAF, Rlc, TCF, Tni, TPT1, Tropomyosin, TROPONIN, Troponin t, TTN and TXNDC17 differentially expressed genes/proteins.

## Discussion

This study aimed to evaluate the genomic and proteomic changes during the early stages of tail tissue regeneration in house lizard. Vertebrates like zebrafish and other invertebrates have been extensively studied for understanding the epimorphic regeneration^34,35^ at the genomic and proteomic level. This study has been performed using the house gecko as the model animal which is similar to our earlier study where we have used other model animals, such as zebrafish^34^ and Echinoderms^35^.

The study not only mapped the transcriptome and proteome map of gecko tail tissue but also identified the list of 254 genes and 128 proteins involved in regeneration of the tail tissue upon amputation during the early stages of regeneration i.e., during 1, 2 and 5-dpa. Several known and unknown genes/proteins which were identified in this study were also involved in epimorphic regeneration of caudal fin tissue of zebrafish^34^; arm of brittle star^35^ and tail of *Hemidactylus flaviviridis*^16^; *Podarcis muralis*^14^. Loss of function through downregulation of genes were majorly observed during regeneration at 1 and 5-dpa rather than gain of function of genes. Whereas at the proteome level it was observed that at 1 and 2-dpa, a prominent gain of function through upregulation of proteins widely. An anomalous downregulation of 67% of the identified proteins at 5-dpa impacts a huge loss of function. Validation of 50 genes identified from the transcriptomic analysis confirms the differential regulation of the genes for the biomechanism of regeneration, which is also evident from the hierarchical heat map analysis of the transcriptome and proteome.

The upregulated gene expression analysis were found associated with muscle tissue development and wound healing processes. Previous research works related to tail regeneration in lizards have listed c-myc upregulation plays a vital role in regeneration mechanism^36^. In thisstudy we have listed BIN1 gene responsible for differentiation^37^ also related to c-myc interaction and expression. This suggests that genes are mostly involved in wound healing phase in the timepoints and presence of BIN1^38^ gene expression might interact with c-myc in later stages. BIN1 is myc-interacting gene which inhibits the expression of c-myc^39^. This could help in optimal expression of c- myc proliferation in further blastemal and regenerating phases. This might indicate controlled expression of c-myc is initiated from preliminary stages and would associate with AEP formation or blastemal stage in latter phase. Also, HOX-D13^40^ gene known for its limb morphogenesis found to be downregulated in all timepoints which interact with shh(another developmental associated gene)^32^ and initiates limb development process. Some other genes like MYOD1, TNNI1, ACTC1 are involved in myogenesis were found to be upregulated in 5-dpa, 2-dpa and downregulated in later timepoints respectively^18^. This association and regulation of genes explains the biological process of initial tail stump processes which would be leading to the blastemal stage of tail regeneration.

Genes like EPB41, RARRES1, PCLAF, ADRM1, Hemoglobin subunit-β (HBB)^41^, Myosin binding proteins-H (MYBPH)^42,43^ and mTORC1 were found upregulated most prominently in the early stages of tail regeneration. Based on Gene ontological biological function RARRES1 is associated with negative regulation of cell proliferation, PCLAF is associated DNA repair and regulation of cell cycle, ADRM1 controls positive regulation of growth hormone receptor signalling pathway and MAP3K7 is associated with interleukin-1 mediated signalling pathway. mTORC1 and ANGPT17 genes were found to be involved in transcriptional regulation, homeostasis, cell growth regulation and metabolism. It has been well known that mTORC1 gain of function leads to neural stem cell differentiation and loss of function associated with limitation of differentiation and neuron production^44^. The downregulated TMBIM6 was found to be associated with autophagy and negative regulation of apoptotic process.

The network/canonical pathways associated with the differentially expressed genes/proteins revealed mostly signalling and metabolic pathways. The major network pathways associated with the tail tissue regeneration are Cell morphology, embryonic development and skin development (Fig. 3b) involving DSC1, DSC2, GPI, DSP, NEDD8, CD63 and keratin as major genes/proteins. NEDD8, a ubiquitin like protein involved in cell cycle progression was found to be associated with the regeneration from 2-dpa onwards through upregulation. CD63, a cell surface receptor plays a key role in activation of AKT (Serin/threonine protein kinase) and integrin leading to cellular signalling cascades was found to be upregulated from day 1 of amputation having an important role in regeneration initiation. Keratinisation, cellular differentiation and epithelial cell proliferation were found to be upregulated for the initiation of regeneration from 2-dpa onwards. FERMT2 (Fermitin family member 2)^45^ and MYO16 were found correlated in cell–cell adhesion, cell junction assembly and regulation of cell cycle progression based on network pathway analysis. These pathways were found associated with epithelial cell differentiation, cellular morphology, proliferation and development, controlling the cell cycle progression, actin-filament binding for structural integrity, cytoskeleton organization and cellular signalling during the tail regeneration.

Similarly, ADRM1, APOA1, ASPH, ATP2A1, PAIP2, SLC24A6 genes/proteins majorly associated with Cellular assembly and organization (Fig. 3c). Role of Pabp-interacting proteins (PAIP) have been shown to be involved in differentiation of adult stem cells in testis through the inhibition of translation^46^. The cellular assembly and organization network pathway (Fig. 3c) were found associated with the immune response (NFKB complex), cell survival, and Integrin-mediated signalling pathways (APOA1)^46^ indicating its association during the initial stages of regeneration. Organ and organismal development network pathway (Fig. 3d) were associated with ACTC1, ACTG1, BIN1, CAPZA2 and CAPZB genes/proteins. This pathway identified the family of actins and cytoskeletal proteins involvement during the development along with translational regulators enabling the mechanism to functionalize in an ordered manner. Skeletal and muscular system development network pathway (Fig. 3e) was associated with CBR1, DLX3, EEF1B2 and DLX3 genes/proteins.

This study has identified and associated various genes/proteins and their network pathways for the tail tissue regeneration of lizard which were also found to be associated with epimorphic regeneration of organs in other animals as in *Podarcis muralis*^14,15,47^, green anole Lizard^18,48^, zebrafish^34^ and echinoderms^35^. The manner of association and dissociation of genes/proteins during tail regeneration in *Hemidactylus frenatus* is attractive to study further. Although detailed understanding of the differential expressed genes/proteins and their regulation might lead to better insight in understanding the regeneration of the gecko tail.This study of transcriptome and proteome profile will benefit future studies in *Hemidactylus frenatus* species.

## Material and methods

### Animal and sample collection

The lizard, *Hemidactylus frenatus* were collected and maintained in a well-ventilated cage with adequate proper diet, optimum temperature (~ 25 °C) and the 12 h light and dark cycle. Amputation of tail tissue were performed on batches of animals (n = 5) in replicates using sterile scalpel blade at 1 cm preceding the distal end of the tail. Regenerating tail tissues preceding the amputation site (3 mm) were collected for each time points i.e., 0-day post amputation (dpa) (control), 1-dpa, 2-dpa and 5-dpa. The amputation and collection of regenerating tissues were performed during the morning hours post feeding (10.00 AM). The collected control and regenerating tail tissues were washed with 1X phosphate-buffered saline (PBS), pooled as batches and snap-frozen in liquid nitrogen and was stored at − 70 °C until use. The animal experiment was performed in accordance with the protocol approved by the Institutional animal ethics committee of Centre for Cellular and Molecular Biology (IAEC/CCMB/Protocol # 66/2014).

### Total RNA isolation

Total RNA was extracted from the pooled tissues of each time points using RNA isoPlus Reagent (Takara Bio, CA, USA) following the manufacturer’s protocol. The RNA yield and purity were calculated using NanoDrop 2000 Thermo fisher and gel analysis.

### NGS transcriptomic analysis

The total RNA transcripts of each time point tissues were obtained based on next generation sequencing (NGS) analysis involving Illumina HiSeq 2000^35^. All the transcripts obtained commonly from all the tissues were further assembled for De novo transcriptome analysis and functional annotation against non-redundant reptilian database using blastx^35^. Both the known and unknown gene sequences obtained from the NGS analysis were tabulated and submitted to NCBI and obtained accession number. Also, the genes were translated for obtaining protein database.

### Differential expression analysis

The transcripts were further analysed for their expression level using FPKM (Fragments per kilobase of transcript per million mapped reads)and analysed for differential expression level on 1, 2 and 5-dpa time points against 0-dpa as control^35^. Differentially expressed transcripts having at least 1.0 log fold change in any one of their regenerating time points were considered for the study. All the differentially expressed genes were analyzed using GENE ONTROLOGY online tool (www.geneontology.org)^49,50^. The gene list weremapped against *Anolis carolinesis* database for biological process. Significant genes having a p value of < 0.05 were selected for all the time points.

### Real Time PCR (RTPCR) analysis

Validation of fifty most significantly expressed genes having more than 2 log fold changes in any of its time points were selected for RTPCR analysis. Primers were designed using Primer3 software(https://bioinfo.ut.ee/primer3-0.4.0/). Amplification of 14-3-3 protein zeta/delta isoform X1 and NADH dehydrogenase 1 alpha sub complex subunit 11 were used as housekeeping gene. RTPCR were performed in biological and technical replicates for each genes from the cDNA synthesized from 1 μg total RNA using Takara SYBR green assay master mix. The relative expressions of the genes were estimated based on the RTPCR Ct value against control (0-dpa) as the baseline.

### Protein extraction and Isobaric tag for relative and absolute quantification(iTRAQ) analysis

The total protein from the control and regenerating tail tissues were extracted using protein extraction buffer (7 M urea, 2 M thiourea, 18 mM Tris–HCl, 4% CHAPS, 14 mM Trizmabase, 2 Tablets EDTA protease Inhibitor, Triton X 0.2%, 50 mM DTT) upon homogenization and sonication^34,51^. The proteins were quantified using Amido black method^52^ against BSA as standard. iTRAQ based quantitative proteomics analysis was performed in duplicates between the control (0-dpa) and regenerating time points (1-dpa, 2-dpa and 5-dpa) by loading 50 μg of total proteins in a 10% SDS-PAGE gel. The gel was stained, destained, documented and fractionated into four sequential groups. The gel fractions were washed; trypsin digested, labelled with isobaric tags iTRAQ 4-plex labelling and purified with the help of C-18 spin columns (Thermo Scientific). The purified/labelled peptides were vacuum dried and constituted in 5% acetonitrile (ACN) and 0.2% formic acid to the peptides for the LCMS/MSMS (Liquid chromatography mass spectrometry/Tandem mass spectrometry) analysis^51^. The LCMS/MSMS run was performed in Orbitrap Velos Nano analyzer (Thermo) involving High Collision Dissociation (HCD) mode of acquisition with 50% normalized collision energy. The raw files were analysed with Sequest HT proteome discoverer 1.4 (Thermo Scientific), with 1% FDR (False discovery rate) using percolator and XCorr (Score Vs Charge) against the NGS generated lizard database. All the proteins, in duplicates, were analysed against the control (0-dpa). Differentially regulated expression by more than 1-log fold changes were selected as proteins associated with regeneration.

### Heat map and network pathway analysis

All the differentially expressed genes and proteins were analysed for the heat map analysis involving heat mapper portal (www.heatmapper.ca) towards elucidating the hierarchical cluster analysis of the genes/proteins and the time points. This analysis helps in visually distinguish the upregulated and downregulated expression of genes involved in all selected time points of regeneration mechanism.The association of these genes / proteins were also analysed for the association in network pathways based on Ingenuity pathway analysis (IPA). This will give an insight on genes association or cross talk within a specific biological processes and its functional roles.

## Supplementary Information

Supplementary Information.
