# Supplementary material for: Transcriptomic and proteomic analysis of Hemidactylus frenatus during initial stages of tail regeneration
Source: Sci Rep. 2021 Feb 11;11:3675. doi: 10.1038/s41598-021-83283-0 (PMC7878758; doi:10.1038/s41598-021-83283-0)
Supplement: Supplementary file 1 — Supplementary Information. [file 41598_2021_83283_MOESM1_ESM.pdf]

Title: Transcriptome and Proteome analysis of *Hemidactylusfrenatus* during initial stages of tail regeneration.

Short title: Regeneration in gecko tail

Sai Pawan, SarenaBanu, Mohammed M Idris*

Sai Pawan, Project based Student, CSIR-CCMB, Uppal Road, Habsiguda, Hyderabad 500007. Email: n.sai.pawan325@gmail.com

SarenaBanu, Project Assisstant, CSIR-CCMB, Uppal Road, Habsiguda, Hyderabad 500007. Email: sarena@ccmb.res.in

Mohammed M Idris, Senior Principal Scientist, CSIR-CCMB, Uppal Road, Habsiguda, Hyderabad 500007. Email: idris@ccmb.res.in

*Corresponding Author

Key words: Transcriptome; Proteome; Regeneration; Gecko; Network pathway

Supplementary Table 1: List of differentially expressed genes based on NGS transcriptomic analysis. Accession, description, symbol, 1dpa fold changes, 2dpa fold changes, 5dpa fold changes, 1dpa p value, 2dpa p values and 5dpa p values were shown in each column.

S. No	Accession	Description	Symbol	Gene Fold Change			p-value		
				1-dpa	2-dpa	5-dpa	1-dpa	2-dpa	5-dpa
**1**	TRINITY_DN30805_c0_g3_i3	Similar to Gekkojaponicus uncharacterized LOC107120650	N/A	-3.7	-3.7	-6.1	**0.0**	**0.0**	0.1
**2**	TRINITY_DN38093_c1_g1_i2	ADAM metallopeptidase domain 9 (ADAM9), mRNA like	ADAM9l	-1.7	-4.6	-6.1	0.2	**0.0**	0.1
**3**	TRINITY_DN24480_c0_g2_i1	3-hydroxybutyrate dehydrogenase, type 2 (BDH2), transcript variant X2, mRNA	BDH2	-1.0	3.6	2.2	1.0	**0.0**	0.5
**4**	TRINITY_DN30186_c0_g1_i6	Prolyl 4-hydroxylase, beta polypeptide (P4HB), transcript variant X1, mRNA	P4HB	-1.0	3.8	5.5	1.0	**0.0**	0.1
**5**	TRINITY_DN19868_c1_g5_i1	Transmembrane protein 128 (TMEM128), mRNA	TMEM128	-0.7	-2.9	-7.4	0.6	0.1	**0.0**
**6**	TRINITY_DN34808_c4_g3_i1	Isolate DB198 basic leucine zipper and W2 domain-containing protein 1 (BZW1) gene	BZW1	-0.7	-3.5	-5.1	0.6	**0.0**	0.1
**7**	TRINITY_DN20768_c1_g1_i1	Ankyrin repeat domain 23 (ANKRD23), mRNA	ANKRD23	-0.4	-3.1	-4.7	0.8	**0.0**	0.1
**8**	TRINITY_DN36827_c0_g1_i3	Uncharacterized	N/A	-0.4	-4.8	-5.3	0.8	**0.0**	0.1
**9**	TRINITY_DN22212_c0_g1_i1	HOXD13 (HoxD13) gene	HOXD13	-0.1	-5.3	-6.9	0.9	**0.0**	**0.0**
**10**	TRINITY_DN26064_c0_g1_i1	FGB gene for beta-fibrinogen,	FGB	-0.1	-7.6	-9.2	0.9	**0.0**	**0.0**
**11**	TRINITY_DN24344_c0_g1_i2	Poly(A) binding protein interacting protein 2	PAIP2	0.0	3.7	2.4	1.0	**0.0**	0.5

		(PAIP2)							
**12**	TRINITY_DN34394_c2_g5_i1	Plakophilin-1-like	PKP1	0.0	-3.4	-4.6	1.0	**0.0**	0.1
**13**	TRINITY_DN24813_c0_g1_i2	Similar to Gekkojaponicus uncharacterized LOC107121858	N/A	0.2	-5.0	-3.6	0.9	**0.0**	0.2
**14**	TRINITY_DN30559_c0_g1_i5	Serine/arginine-rich splicing factor 2 (SRSF2)	SRSF2	0.6	3.4	3.2	0.7	**0.0**	0.3
**15**	TRINITY_DN34182_c1_g1_i1	Myosin-4-like	MYH4	0.9	-1.3	-8.5	0.5	0.4	**0.0**
**16**	TRINITY_DN27889_c0_g3_i1	Peroxisome proliferator-activated receptor alpha (PPARA)	PPARA	0.9	-4.4	-4.0	0.5	**0.0**	0.2
**17**	TRINITY_DN26747_c2_g1_i4	Similar to Gekkojaponicus uncharacterized LOC107111273	N/A	0.9	-5.9	-1.9	0.5	**0.0**	0.5
**18**	TRINITY_DN19823_c3_g3_i1	Similar to Gekkojaponicus uncharacterized LOC107117390	N/A	1.0	-5.5	-5.1	0.5	**0.0**	0.1
**19**	TRINITY_DN30281_c0_g4_i1	Prosaposin-like	PSAP	1.1	-5.1	-6.7	0.4	**0.0**	0.1
**20**	TRINITY_DN20187_c0_g2_i1	Serine peptidase inhibitor, Kunitz type 1 (SPINT1)	SPINT1	1.2	-3.3	-3.9	0.4	**0.0**	0.2
**21**	TRINITY_DN27754_c0_g2_i2	Cytosolic arginine sensor for mTORC1 subunit 1	mTORC1	3.9	2.3	-0.6	**0.0**	0.3	1.0
**22**	TRINITY_DN26638_c0_g3_i1	Angiopoietin like 7 (angptl7)	angptl7	4.0	3.0	-0.6	**0.0**	0.1	1.0
**23**	TRINITY_DN36464_c1_g1_i2	Chromosome unknown open reading frame, human C8orf22 like	C8orf22l	4.8	3.9	-0.6	**0.0**	**0.0**	1.0
**24**	TRINITY_DN23291_c3_g5_i1	Uncharacterized	N/A	7.8	8.3	4.3	**0.0**	**0.0**	0.2
**25**	TRINITY_DN38002_c1_g2_i2	Calsequestrin-1	CASQ1	-7.1	-1.6	-8.6	**0.0**	0.3	**0.0**
**26**	TRINITY_DN22817_c0_g2_i5	myosin-1B-like isoform X4	MYO1B	-6.5	-3.0	-5.7	**0.0**	0.1	0.1
**27**	TRINITY_DN32422_c0_g2_i3	myosin-1B-like isoform	MYO1B	-5.9	-2.3	-3.0	**0.0**	0.1	0.3

		X8							
**28**	TRINITY_DN30722_c0_g2_i4	muscle-related coiled-coil protein	MURC	-3.9	-1.9	-2.9	**0.0**	0.2	0.3
**29**	TRINITY_DN34854_c2_g1_i3	UAP56-interacting factor-like	FYTTD1	-3.9	-1.1	-1.7	**0.0**	0.5	0.5
**30**	TRINITY_DN30393_c0_g2_i1	keratin, type II cytoskeletal 4-like	KRT4	-3.5	-4.3	-4.1	**0.0**	**0.0**	0.2
**31**	TRINITY_DN38213_c1_g1_i5	MEF2-activating motif and SAP domain-containing transcriptional regulator	MEF2	-2.7	-3.7	-4.3	0.1	**0.0**	0.2
**32**	TRINITY_DN22052_c0_g2_i6	osteocalcin	BGLAP	-2.7	-4.0	-4.0	0.1	**0.0**	**0.0**
**33**	TRINITY_DN36268_c0_g1_i2	myomegalin isoform X2	pde4dip	-2.2	-3.8	-4.4	0.1	**0.0**	0.2
**34**	TRINITY_DN38093_c1_g1_i3	disintegrin and metalloproteinase domain-containing protein 9	ADAM9	-2.2	-3.6	-1.5	0.1	**0.0**	0.6
**35**	TRINITY_DN36578_c0_g1_i4	ryanodine receptor 2	RYR2	-2.2	-4.2	-4.8	0.1	**0.0**	0.1
**36**	TRINITY_DN26958_c0_g3_i1	homeobox protein DLX-3	DLX3	-2.1	-3.4	-3.4	0.1	**0.0**	0.1
**37**	TRINITY_DN37846_c0_g1_i3	desmoplakin isoform X1	DSP	-3.7	-4.0	-4.6	**0.0**	**0.0**	0.2
**38**	TRINITY_DN22305_c3_g3_i2	NEDD8	NEDD8	-1.6	3.5	3.6	0.6	**0.0**	0.2
**39**	TRINITY_DN38176_c1_g2_i2	uricase-like	UOX	-1.5	-5.1	-5.1	0.3	**0.0**	**0.0**
**40**	TRINITY_DN22220_c0_g1_i1	afadin isoform X6	AFDN	-1.4	-3.7	-4.3	0.3	**0.0**	0.2
**41**	TRINITY_DN38238_c1_g1_i1	nebulin isoform X12	NEB	-1.3	-6.2	-4.3	0.3	**0.0**	**0.0**
**42**	TRINITY_DN28089_c0_g3_i1	vesicle-associated membrane protein-associated protein B/C	VAPB	-1.2	-3.8	-4.4	0.4	**0.0**	0.2
**43**	TRINITY_DN25660_c0_g5_i1	17-beta-hydroxysteroid dehydrogenase 14-like	HSD17B14	-3.2	-5.8	-7.4	**0.0**	**0.0**	**0.0**
**44**	TRINITY_DN36527_c0_g2_i1	death-associated protein-like 1	DAPL1	-0.8	-3.8	-0.8	0.6	**0.0**	0.1
**45**	TRINITY_DN31814_c0_g2_i2	leukocyte elastase inhibitor-like	SERPINB1	-0.7	-6.2	-0.7	0.6	**0.0**	**0.0**
**46**	TRINITY_DN21431_c0_g2_i3	poly(rC)-binding protein 2	PCBP2	-0.3	3.8	3.9	1.0	**0.0**	0.2

		isoform X11							
**47**	TRINITY_DN26467_c0_g1_i1	NHP2-like protein 1	SNU13	0.0	4.0	3.8	1.0	**0.0**	0.2
**48**	TRINITY_DN37657_c1_g4_i2	p53 apoptosis effector related to PMP-22	PMP-22	0.0	-3.9	-4.5	1.0	**0.0**	0.2
**49**	TRINITY_DN35820_c0_g1_i2	desmin	DES	0.4	-2.5	-7.3	0.8	0.1	**0.0**
**50**	TRINITY_DN26831_c0_g2_i3	shematrin-like protein 2	SHEM1	0.6	-6.8	-7.8	0.7	**0.0**	**0.0**
**51**	TRINITY_DN34867_c2_g1_i3	methyltransferase-like protein 7A	METTL7A	1.3	-3.8	-4.4	0.3	**0.0**	0.2
**52**	TRINITY_DN32563_c0_g2_i2	DDB1- and CUL4-associated factor 6 isoform X1	DCAF6	1.5	3.5	3.2	0.3	**0.0**	0.3
**53**	TRINITY_DN19443_c0_g2_i1	C-C motif chemokine 5-like	CCL5	1.9	3.7	2.2	0.2	**0.0**	0.5
**54**	TRINITY_DN27678_c0_g3_i1	retinol-binding protein 5	RBP5	2.2	3.4	1.9	0.1	**0.0**	0.5
**55**	TRINITY_DN36609_c1_g2_i3	mitogen-activated protein kinase kinasekinase 7 isoform X2	MAP3K7	3.1	2.7	2.2	**0.0**	0.1	0.5
**56**	TRINITY_DN35430_c0_g1_i4	proteasomal ubiquitin receptor ADRM1	ADRM1	3.5	3.6	3.6	**0.0**	0.1	0.8
**57**	TRINITY_DN23823_c0_g2_i4	AN1-type zinc finger protein 2A	ZFAND2A	3.6	3.0	3.0	**0.0**	0.1	0.8
**58**	TRINITY_DN23648_c1_g2_i1	cathelicidin-related peptide Oh-Cath-like	CATH	3.7	4.8	-0.6	**0.0**	**0.0**	1.0
**59**	TRINITY_DN24620_c0_g5_i1	phospholipase A2 inhibitor subunit gamma B-like	PLA2	3.9	4.4	1.3	**0.0**	**0.0**	0.7
**60**	TRINITY_DN36845_c0_g1_i4	alpha-actinin-3	ACTN3	4.9	5.1	2.4	**0.0**	**0.0**	0.4
**61**	TRINITY_DN31052_c0_g1_i2	retinoic acid receptor responder protein 1	RARRES1	5.0	2.9	1.0	**0.0**	0.1	0.8
**62**	TRINITY_DN23991_c0_g2_i1	PCNA-associated factor	PCLAF	3.9	3.9	5.2	1.0	**0.0**	0.1
**63**	TRINITY_DN27770_c0_g4_i1	14-3-3 protein sigma	SFN	-1.6	-5.7	-5.3	0.2	**0.0**	0.1
**64**	TRINITY_DN20642_c2_g2_i3	39S ribosomal protein L17, mitochondrial	MRPL17	-3.9	1.3	2.3	**0.0**	0.4	0.4

**65**	TRINITY_DN24484_c0_g4_i1	40S ribosomal protein S11	RPS11	-3.5	1.0	1.7	**0.0**	0.5	0.5
**66**	TRINITY_DN26256_c1_g1_i6	40S ribosomal protein S12	RPS12	-0.2	0.9	-8.3	0.9	0.5	**0.0**
**67**	TRINITY_DN20830_c0_g5_i1	40S ribosomal protein S24 isoform X2	RPS24	0.1	0.8	-8.6	1.0	0.6	**0.0**
**68**	TRINITY_DN23044_c0_g1_i1	40S ribosomal protein S25	RPS25	-3.0	0.9	1.8	**0.0**	0.5	0.5
**69**	TRINITY_DN24077_c0_g2_i3	60S ribosomal protein L18	RPL18	-3.7	1.3	1.8	**0.0**	0.4	0.5
**70**	TRINITY_DN20257_c0_g1_i4	60S ribosomal protein L35a	RPL35A	0.2	1.6	-7.4	0.9	0.3	**0.0**
**71**	TRINITY_DN35974_c0_g2_i1	A.superbus venom factor 1-like	AVF1	0.0	3.6	3.6	1.0	**0.0**	0.2
**72**	TRINITY_DN31002_c0_g2_i2	actin, alpha cardiac muscle 1	ACTC1	0.8	-0.9	-8.0	0.6	0.5	**0.0**
**73**	TRINITY_DN35992_c0_g2_i1	adenylosuccinatesynthetase isozyme 1	ADSSL1	-1.5	-3.4	-3.0	0.3	**0.0**	0.3
**74**	TRINITY_DN37906_c2_g1_i5	alpha-enolase isoform X1	ENO1	-3.9	0.1	-0.6	**0.0**	1.0	0.8
**75**	TRINITY_DN34147_c2_g1_i3	alpha-sarcoglycan isoform X2	SGCA	-3.2	0.3	-0.2	**0.0**	0.9	1.0
**76**	TRINITY_DN34179_c3_g3_i1	annexin A2	ANXA2	-0.5	0.3	-8.5	0.7	0.9	**0.0**
**77**	TRINITY_DN25783_c2_g1_i5	aspartyl/asparaginyl beta-hydroxylase isoform X3	ASPH	-3.8	1.4	0.1	**0.0**	0.4	1.0
**78**	TRINITY_DN30115_c0_g2_i10	ATP synthase F(0) complex subunit C1, mitochondrial	ATP5MC1	-4.3	0.8	1.5	**0.0**	0.6	0.6
**79**	TRINITY_DN26389_c0_g5_i1	ATP synthase F(0) complex subunit C2, mitochondrial	ATP5MC2	-3.6	1.1	1.0	**0.0**	0.5	0.7
**80**	TRINITY_DN22271_c0_g2_i2	ATP synthase subunit d, mitochondrial	ATP5PD	-4.1	0.9	2.3	**0.0**	0.5	0.4
**81**	TRINITY_DN21311_c1_g1_i1	avidin-like	AVD	3.1	2.5	4.7	**0.0**	0.1	0.1
**82**	TRINITY_DN33064_c0_g1_i3	bax inhibitor 1	TMBIM6	-4.5	1.0	0.8	**0.0**	0.5	0.8
**83**	TRINITY_DN25564_c1_g2_i5	bcl-2-like protein 10	BCL2L10	3.5	2.3	0.0	**0.0**	0.2	1.0
**84**	TRINITY_DN32628_c1_g1_i1	beta-2-microglobulin	B2M	-0.4	-0.3	-6.7	0.8	0.8	**0.0**

		isoform X1							
**85**	TRINITY_DN37021_c1_g1_i1	beta-enolase	ENO3	-0.7	-1.0	-8.5	0.7	0.5	**0.0**
**86**	TRINITY_DN29143_c0_g1_i3	betaine--homocysteine S-methyltransferase 1	BHMT	3.8	2.0	-2.9	**0.0**	0.2	0.4
**87**	TRINITY_DN32939_c2_g1_i1	CCAAT/enhancer-binding protein delta	CEBPD	-3.5	-1.1	1.2	**0.0**	0.4	0.7
**88**	TRINITY_DN32564_c0_g3_i6	CD63 antigen	CD63	-3.6	1.2	1.7	**0.0**	0.4	0.5
**89**	TRINITY_DN24309_c1_g1_i2	claw keratin-like	CKER1	3.6	-7.5	-4.1	**0.0**	**0.0**	0.2
**90**	TRINITY_DN27033_c0_g1_i5	cold-inducible RNA-binding protein isoform X1	CIRBP	-5.4	-0.2	-0.2	**0.0**	0.9	0.9
**91**	TRINITY_DN37825_c1_g3_i1	collagen alpha-1(I) chain	COL1A1	-1.3	-4.4	-7.8	0.4	**0.0**	**0.0**
**92**	TRINITY_DN34178_c2_g1_i2	creatine kinase M-type	CKM	-6.0	-0.3	-0.8	**0.0**	0.8	0.7
**93**	TRINITY_DN37492_c0_g1_i3	C-type lectin domain family 2 member D-like isoform X1	CLEC2D	-4.5	-0.6	-0.2	**0.0**	0.7	1.0
**94**	TRINITY_DN19561_c3_g3_i1	cytochrome c oxidase assembly factor 5	COA5	-3.7	0.9	1.8	**0.0**	0.5	0.5
**95**	TRINITY_DN30220_c3_g2_i1	cytochrome c oxidase subunit I (mitochondrion)	MT-CO1	-10.7	1.0	0.9	**0.0**	0.4	0.7
**96**	TRINITY_DN25546_c6_g2_i1	cytochrome c oxidase subunit II (mitochondrion)	MT-CO2	-9.2	1.9	2.2	**0.0**	0.2	0.4
**97**	TRINITY_DN31796_c0_g2_i4	cytosolic phospholipase A2 gamma	PLA2G4C	-1.2	-3.8	-4.4	0.4	**0.0**	0.2
**98**	TRINITY_DN34475_c0_g4_i2	dehydrogenase/reductase SDR family member 7C	DHRS7C	-3.8	-0.8	-2.1	**0.0**	0.6	0.4
**99**	TRINITY_DN27718_c1_g2_i1	desmin like	DES	-3.9	-0.7	-1.6	**0.0**	0.6	0.6
**100**	TRINITY_DN37400_c1_g1_i7	desmocollin-1 isoform X1	DSC1	-0.2	-4.1	-4.7	0.9	**0.0**	0.1
**101**	TRINITY_DN38178_c1_g2_i3	DNA-binding death effector domain-containing protein 2	DEDD2	-3.6	-0.8	0.0	**0.0**	0.6	1.0
**102**	TRINITY_DN25885_c3_g1_i1	E3 ubiquitin-protein ligase TRIM63	TRIM63	3.0	4.8	-2.0	**0.0**	**0.0**	0.5

**103**	TRINITY_DN28739_c2_g1_i1	elongation factor 1-beta	EEF1B2	-4.5	2.2	2.2	**0.0**	0.1	0.4
**104**	TRINITY_DN36346_c1_g1_i2	endoplasmic reticulum junction formation protein lunapark isoform X1	Lnpk	-3.7	-0.4	-0.7	**0.0**	0.8	0.8
**105**	TRINITY_DN29939_c0_g1_i1	epididymal secretory protein E1	NPC2	2.9	1.4	-2.2	**0.0**	0.4	0.6
**106**	TRINITY_DN20765_c0_g1_i1	ETS-related transcription factor Elf-3	ELF3	1.6	5.0	1.0	0.6	**0.0**	1.0
**107**	TRINITY_DN37821_c1_g1_i2	eukaryotic initiation factor 4A-II	EIF4A2	-2.8	-0.1	-0.2	**0.0**	1.0	0.9
**108**	TRINITY_DN33119_c0_g1_i3	fatty acid desaturase 1-like	FADS1	1.6	5.1	-0.6	0.6	**0.0**	1.0
**109**	TRINITY_DN33871_c5_g3_i3	feather keratin B-4-like	FKER4	0.1	-4.3	-9.3	1.0	**0.0**	**0.0**
**110**	TRINITY_DN27663_c1_g2_i5	ferritin, higher subunit-like	FTL	-7.0	0.3	1.6	**0.0**	0.8	0.6
**111**	TRINITY_DN22711_c0_g2_i1	fish-egg lectin-like	FEL	-3.5	0.6	0.5	**0.0**	0.7	0.9
**112**	TRINITY_DN32810_c4_g1_i6	fructose-bisphosphate aldolase A	ALDA	-3.7	-1.7	2.9	**0.0**	0.2	0.3
**113**	TRINITY_DN28408_c0_g2_i2	glutamine synthetase	GLUL	3.0	1.2	-4.8	**0.0**	0.4	0.1
**114**	TRINITY_DN37527_c4_g3_i4	glyceraldehyde-3-phosphate dehydrogenase	GAPDH	-5.2	0.8	0.4	**0.0**	0.3	0.9
**115**	TRINITY_DN33097_c1_g3_i1	glycerol-3-phosphate dehydrogenase [NAD(+)], cytoplasmic	GAPD1	-3.3	0.3	-0.1	**0.0**	0.8	1.0
**116**	TRINITY_DN27794_c1_g1_i3	glycine-rich cell wall structural protein-like	GRP1	0.9	-4.6	-6.2	0.5	**0.0**	0.1
**117**	TRINITY_DN37554_c1_g1_i3	glycogen phosphorylase, liver form	PYGL	0.4	-2.6	-7.5	0.8	0.1	**0.0**
**118**	TRINITY_DN37554_c1_g1_i2	glycogen phosphorylase, muscle form	PYGM	-4.3	-3.7	-4.9	**0.0**	**0.0**	0.1
**119**	TRINITY_DN30693_c1_g1_i3	GTPaseHRas-like	HRAS	-3.6	0.2	0.5	**0.0**	0.9	0.9
**120**	TRINITY_DN28822_c3_g3_i1	heat shock cognate 71 kDa protein-like	HSPA8	-3.8	0.4	0.4	**0.0**	0.8	0.9
**121**	TRINITY_DN38168_c1_g1_i1	heat shock protein 30C-	HSP30C	2.9	-0.8	-4.8	**0.0**	0.6	0.1

		like							
**122**	TRINITY_DN33508_c0_g1_i3	heterogeneous nuclear ribonucleoprotein H isoform X4	HNRNPH1	2.7	4.0	3.4	0.1	**0.0**	0.2
**123**	TRINITY_DN19565_c0_g2_i1	HIG1 domain family member 2A, mitochondrial	HIG2A	-3.3	1.2	1.8	**0.0**	0.4	0.5
**124**	TRINITY_DN23441_c0_g3_i6	histidine triad nucleotide-binding protein 1	HINT1	-3.7	0.8	1.5	**0.0**	0.6	0.6
**125**	TRINITY_DN35958_c1_g1_i7	inosine-5'-monophosphate dehydrogenase 1	IMPDH1	-3.5	-0.5	-1.0	**0.0**	0.8	0.7
**126**	TRINITY_DN35280_c0_g1_i2	keratin, type I cytoskeletal 10-like	KRT10	0.4	-4.1	-4.7	0.8	**0.0**	0.1
**127**	TRINITY_DN37110_c0_g1_i3	keratin, type I cytoskeletal 14-like	KRT14	2.3	4.8	-1.2	0.1	**0.0**	0.8
**128**	TRINITY_DN20271_c0_g1_i2	keratin, type I cytoskeletal 19	KRT19	-2.7	-5.2	-7.2	0.1	**0.0**	**0.0**
**129**	TRINITY_DN34028_c0_g1_i5	keratin, type I cytoskeletal 23-like	KRT23	0.2	-3.7	-5.6	0.9	**0.0**	0.1
**130**	TRINITY_DN20247_c0_g2_i3	keratin, type I cytoskeletal 24-like	KRT24	-0.1	-4.8	-7.4	1.0	**0.0**	**0.0**
**131**	TRINITY_DN37419_c0_g2_i1	keratin, type II cytoskeletal 5-like	KRT5	-3.0	-3.4	-7.0	**0.0**	**0.0**	**0.0**
**132**	TRINITY_DN37980_c1_g3_i3	keratin, type II cytoskeletal cochleal-like	KRT7	-0.3	-5.7	-9.0	0.8	**0.0**	**0.0**
**133**	TRINITY_DN33770_c1_g2_i1	keratin-associated protein 5-5-like	KRTAP5-5	0.4	-3.9	-0.5	0.8	**0.0**	0.9
**134**	TRINITY_DN21461_c0_g2_i2	keratin-associated protein 9-1-like	KRTAP9-1	0.7	-4.3	-2.7	0.6	**0.0**	0.3
**135**	TRINITY_DN24317_c2_g1_i3	luc7-like protein 3 isoform X1	LUC7L3	-4.0	1.0	1.3	**0.0**	0.5	0.6
**136**	TRINITY_DN30544_c0_g1_i3	ly6/PLAUR domain-containing protein 3-like	LYPD3	0.2	-5.6	-2.3	0.9	**0.0**	0.4
**137**	TRINITY_DN30209_c0_g2_i1	lymphocyte antigen 6E-	Ly6a	0.4	-3.7	-2.7	0.8	**0.0**	0.3

		like							
**138**	TRINITY_DN29603_c0_g1_i4	MAPK regulated corepressor interacting protein 2	MCRIP2	2.9	2.1	-1.6	**0.0**	0.2	0.7
**139**	TRINITY_DN26604_c2_g2_i6	microtubule-associated proteins 1A/1B light chain 3B	MAP1LC3B	3.7	1.0	-0.6	**0.0**	1.0	1.0
**140**	TRINITY_DN35142_c1_g2_i2	mitochondrial uncoupling protein 3	UCP3	1.2	4.0	-2.6	0.5	**0.0**	0.5
**141**	TRINITY_DN34043_c1_g1_i3	myc box-dependent-interacting protein 1 isoform X6	BIN1	-2.9	-0.6	-1.1	**0.0**	0.7	0.7
**142**	TRINITY_DN37316_c3_g1_i4	myc box-dependent-interacting protein 1 isoform X8	BIN1	-5.0	1.0	0.9	**0.0**	0.5	0.7
**143**	TRINITY_DN31785_c1_g2_i6	myelin P2 protein-like	PMP2	2.3	-4.2	-4.8	0.1	**0.0**	0.1
**144**	TRINITY_DN30564_c1_g1_i4	myoblast determination protein 1	MYOD1	-3.3	0.5	1.0	**0.0**	0.7	0.7
**145**	TRINITY_DN38080_c1_g1_i2	myomesin-1 isoform X1	MYOM1	-2.1	-3.7	-4.3	0.2	**0.0**	0.2
**146**	TRINITY_DN34011_c0_g1_i1	myosin heavy chain, skeletal muscle, adult	MYH1	-1.0	-4.6	-7.0	0.5	**0.0**	**0.0**
**147**	TRINITY_DN34714_c1_g1_i2	myosin light chain 1/3, skeletal muscle isoform isoform X2	MYL1	-5.3	0.8	0.1	**0.0**	0.5	1.0
**148**	TRINITY_DN23319_c0_g1_i6	myosin light polypeptide 6 isoform X2	MYL6	-3.2	0.7	1.2	**0.0**	0.7	0.6
**149**	TRINITY_DN23306_c0_g2_i1	myosin regulatory light chain 2, ventricular/cardiac muscle isoform	MYL2	-0.5	-4.4	1.1	0.7	**0.0**	0.7
**150**	TRINITY_DN38338_c2_g2_i3	myosin-1B-like isoform X2	MYO1B	-0.9	-3.8	-6.2	0.5	**0.0**	0.1
**151**	TRINITY_DN38343_c2_g1_i2	myosin-1B-like isoform X4	MYO1B	0.2	-0.8	-6.7	0.9	0.6	**0.0**

**152**	TRINITY_DN38338_c1_g1_i3	myosin-1B-like isoform X4 like	MYO1B	-3.1	-3.3	-2.5	**0.0**	**0.0**	0.4
**153**	TRINITY_DN26737_c2_g1_i1	myosin-3-like isoform X7	MYH3	-5.5	1.2	0.5	**0.0**	0.3	0.9
**154**	TRINITY_DN30527_c1_g1_i1	myosin-7B isoform X2	MYH7B	-3.0	1.1	0.1	**0.0**	0.5	1.0
**155**	TRINITY_DN37664_c0_g1_i2	myosin-binding protein C, fast-type	MYBPC2	-3.6	0.1	-0.1	**0.0**	1.0	1.0
**156**	TRINITY_DN32432_c1_g1_i3	myozenin-1 isoform X1	MYOZ1	-3.6	0.3	0.0	**0.0**	0.9	1.0
**157**	TRINITY_DN21082_c0_g1_i3	NADH dehydrogenase [ubiquinone] 1 subunit C2	NDUFC2	-5.1	1.2	1.8	**0.0**	0.4	0.5
**158**	TRINITY_DN21927_c2_g5_i1	NADH dehydrogenase subunit 5 (mitochondrion)	NDUFV2	5.2	4.7	1.7	**0.0**	**0.0**	0.6
**159**	TRINITY_DN38263_c1_g1_i3	nebulin isoform X34	NEB	-0.9	-3.7	-4.8	0.5	**0.0**	0.1
**160**	TRINITY_DN16072_c1_g1_i3	Similar to Apteryx australismantelli genome assembly AptMant0	N/A	-4.0	-1.1	-1.3	**0.0**	0.5	0.6
**161**	TRINITY_DN18856_c0_g1_i1	Scavenger receptor class B member 2 S homeolog (scarb2.S), mRNA	SCARB2	2.0	4.0	1.0	0.4	**0.0**	1.0
**162**	TRINITY_DN18900_c1_g6_i1	Keratin-associated protein 4-8-like (LOC107109571), mRNA	KRTAP4-8	4.2	2.0	1.0	**0.0**	0.4	1.0
**163**	TRINITY_DN20555_c2_g2_i3	Zinc finger protein 239-like (LOC107181380), mRNA	ZFP239	2.8	3.3	3.7	0.1	**0.0**	0.2
**164**	TRINITY_DN20594_c5_g1_i3	Similar to Scarelusanthracinus voucher UPOL NG0053 NADH dehydrogenase subunit 5 (ND5) gene	ND5	-4.1	0.4	1.2	**0.0**	0.8	0.7
**165**	TRINITY_DN20871_c1_g2_i1	Cathepsin Z (CTSZ)	CTSZ	0.7	-4.5	-5.7	0.6	**0.0**	0.1
**166**	TRINITY_DN20937_c1_g4_i2	Solute carrier family 38 member 5 (SLC38A5)	SLC38A5	-3.7	1.4	1.8	**0.0**	0.3	0.5

**167**	TRINITY_DN21620_c1_g1_i3	Monocarboxylate transporter 1-like (LOC107125559), mRNA	SLC16A1	3.5	2.0	-0.6	**0.0**	0.4	1.0
**168**	TRINITY_DN22245_c1_g1_i2	Cyclic AMP-dependent transcription factor ATF-2	ATF2	-0.6	3.5	2.2	1.0	**0.0**	0.5
**169**	TRINITY_DN23291_c3_g7_i1	Calsequestrin-1 like	CASQ1	-8.4	0.3	0.1	**0.0**	0.9	1.0
**170**	TRINITY_DN23323_c1_g1_i1	Parvalbumin (PVALB), mRNA	PVALB	3.0	1.6	-3.8	**0.0**	0.3	0.3
**171**	TRINITY_DN23920_c1_g2_i2	WD repeat and SOCS box containing 1 (WSB1),	WB1	-5.2	1.1	1.4	**0.0**	0.4	0.6
**172**	TRINITY_DN24078_c2_g1_i1	Ring finger protein 24 (RNF24), transcript variant X1, mRNA	RNF24	1.4	-4.3	-4.9	0.3	**0.0**	0.1
**173**	TRINITY_DN24197_c1_g1_i1	KIS(5-71) SINE Squam1, complete sequence	Squam1	3.9	-1.0	0.7	**0.0**	1.0	0.9
**174**	TRINITY_DN24202_c0_g4_i2	Similar to Gekkojaponicus sarcolipin (SLN), transcript variant X1	SLN	-8.5	1.0	-0.6	**0.0**	0.5	0.8
**175**	TRINITY_DN24910_c2_g3_i9	Plasmolipin (PLLP), mRNA	PLLP	2.9	0.4	-2.2	**0.0**	1.0	0.6
**176**	TRINITY_DN26094_c0_g2_i6	Similar to Cyprinuscarpio genome assembly common carp genome	N/A	0.7	-5.8	-2.1	0.6	**0.0**	0.4
**177**	TRINITY_DN26203_c0_g1_i1	Similar to Drosophila ficusphila sodium/potassium/calcium exchanger Nckx30C	Nckx30C	-4.2	1.0	0.4	**0.0**	0.5	0.9
**178**	TRINITY_DN26440_c3_g2_i2	Erythrocyte membrane protein band 4.1 (EPB41), transcript variant X13, mRNA	EPB41	6.0	3.7	-0.6	**0.0**	**0.0**	1.0
**179**	TRINITY_DN26576_c1_g1_i1	DOT1 like histone lysine methyltransferase	DOT1L	-3.8	1.1	1.6	**0.0**	0.4	0.6

		(DOT1L)							
**180**	TRINITY_DN27369_c1_g4_i1	Ring finger protein 20 (RNF20), mRNA	RNF20	-4.2	1.5	2.5	**0.0**	0.3	0.4
**181**	TRINITY_DN27386_c1_g1_i4	ATPase inhibitory factor 1 (ATPIF1)	ATPIF1	-5.2	0.9	1.8	**0.0**	0.5	0.5
**182**	TRINITY_DN27804_c1_g2_i1	Glutamate receptor interacting protein 2	GRIP2	0.0	4.2	0.0	1.0	**0.0**	1.0
**183**	TRINITY_DN27804_c1_g2_i5	Solute carrier family 6 (neurotransmitter transporter), member 6 (SLC6A6), transcript variant X3, mRNA	SLC6A6	3.3	0.7	-2.2	**0.0**	0.8	0.6
**184**	TRINITY_DN27900_c1_g1_i3	Premature ovarian failure, 1B (POF1B)	POF1B	-4.6	1.4	2.1	**0.0**	0.4	0.4
**185**	TRINITY_DN27971_c0_g2_i2	Lymphocyte antigen 86 (LY86), transcript variant X1, mRNA	LY86	3.5	-0.6	-2.2	**0.0**	1.0	0.6
**186**	TRINITY_DN28000_c3_g1_i2	28S ribosomal RNA	RPS28	-4.3	0.4	0.3	**0.0**	0.8	0.9
**187**	TRINITY_DN28023_c1_g1_i1	Xin actin-binding repeat-containing protein 2-like	XIRP2l	-6.8	0.8	1.3	**0.0**	0.6	0.6
**188**	TRINITY_DN28063_c0_g2_i3	Myozenin 3 (MYOZ3)	MYOZ3	-3.7	-0.1	-1.7	**0.0**	1.0	0.5
**189**	TRINITY_DN28124_c0_g5_i6	Tropomyosin 1 (TPM1), transcript variant X5,	TPM1	-5.3	-0.1	-0.4	**0.0**	0.9	0.9
**190**	TRINITY_DN29368_c2_g1_i1	similar to Podarcismuralis uncharacterized LOC114586692 (LOC114586692), ncRNA	LOC114586692	1.3	-5.0	-7.1	0.4	**0.0**	**0.0**
**191**	TRINITY_DN30390_c1_g1_i1	Myc box-dependent-interacting protein 1	BIN1	-4.1	0.3	0.3	**0.0**	0.9	0.9
**192**	TRINITY_DN30395_c0_g1_i5	Keratin, type II cytoskeletal 1	KRT1	-3.5	-3.2	-7.1	**0.0**	**0.0**	**0.0**
**193**	TRINITY_DN30509_c0_g1_i2	Calcium/calmodulin dependent protein kinase II	CAMK2G	-3.5	0.3	0.3	**0.0**	0.9	0.9

		gamma (CAMK2G)							
**194**	TRINITY_DN30512_c2_g1_i4	Fermitin family member 2 (FERMT2), transcript variant X4, mRNA	FERMT2	3.5	2.3	-0.6	**0.0**	0.3	1.0
**195**	TRINITY_DN30587_c0_g2_i1	Uncharacterized	N/A	-2.9	1.2	1.6	**0.0**	0.4	0.6
**196**	TRINITY_DN30884_c0_g2_i1	FANCD2/FANCI-associated nuclease 1 (FAN1)	FAN1	-4.7	0.9	1.1	**0.0**	0.5	0.7
**197**	TRINITY_DN31847_c0_g3_i1	Cold inducible RNA binding protein	CIRBP	-3.7	0.5	1.5	**0.0**	0.7	0.6
**198**	TRINITY_DN32104_c3_g1_i3	Integrin subunit beta 2 (ITGB2), mRNA	ITGB2	3.3	-3.7	-4.3	**0.0**	**0.0**	0.2
**199**	TRINITY_DN32564_c0_g2_i1	CD63 molecule (CD63), transcript variant X2, mRNA	CD63	3.2	2.5	1.2	**0.0**	0.1	0.8
**200**	TRINITY_DN32941_c3_g1_i2	Triosephosphate isomerase 1 (TPI1), mRNA	TPI1	1.5	1.0	-6.9	0.3	0.5	**0.0**
**201**	TRINITY_DN33871_c5_g3_i1	Similar to Hemidactylusturcicus mRNA for ge-gprp4	gpr4	-0.4	-3.8	2.1	0.8	**0.0**	0.5
**202**	TRINITY_DN34416_c0_g3_i1	Centromere-associated protein E (LOC108000233), transcript variant X2	CENPE	-4.1	0.7	1.2	**0.0**	0.7	0.7
**203**	TRINITY_DN34551_c1_g1_i2	Unconventional myosin-XVI-like	MYO16	-4.8	1.9	0.1	**0.0**	0.2	1.0
**204**	TRINITY_DN35421_c1_g1_i4	Xin actin binding repeat containing 2 (XIRP2)	XIRP2	-4.0	-0.3	-1.1	**0.0**	0.8	0.7
**205**	TRINITY_DN36311_c0_g2_i1	Homocysteine-inducible, endoplasmic reticulum stress-inducible, ubiquitin-like domain member 1 (HERPUD1)	HERPUD1	-3.5	-0.1	1.3	**0.0**	1.0	0.6

**206**	TRINITY_DN37045_c0_g1_i3	Unknown open reading frame, human C8orf22 (LOC107107199), mRNA	C8orf22	4.5	2.8	-2.6	**0.0**	0.1	0.5
**207**	TRINITY_DN37251_c1_g5_i2	Calpain small subunit 1	CAPNS1	-3.5	0.6	1.7	**0.0**	0.7	0.5
**208**	TRINITY_DN37466_c1_g3_i1	NDRG1 protein	NDRG1	-1.0	3.8	0.4	1.0	**0.0**	1.0
**209**	TRINITY_DN37517_c6_g2_i2	Parvalbumin beta-like (LOC107123052), mRNA	N/A	3.4	3.4	1.8	0.1	**0.0**	0.5
**210**	TRINITY_DN37631_c0_g1_i1	Similar to Gopherusevgoodei uncharacterized LOC115654989	N/A	-5.2	0.3	0.4	**0.0**	0.9	0.9
**211**	TRINITY_DN37929_c1_g1_i4	Methylthioadenosine phosphorylase (MTAP)	MTAP	-7.3	0.2	-1.2	**0.0**	0.9	0.7
**212**	TRINITY_DN38030_c0_g2_i3	Autophagy-related protein 9A-like (LOC107118613), transcript variant X6, mRNA	ATG9A	3.0	1.3	-1.6	**0.0**	0.4	0.7
**213**	TRINITY_DN38294_c1_g2_i2	Periostin (POSTN), transcript variant X12	POSTN	-3.0	1.6	0.9	**0.0**	0.3	0.7
**214**	TRINITY_DN38304_c1_g2_i1	Collagen type III alpha 1 chain (COL3A1)	COL3A1	-1.6	-5.4	-0.1	0.2	**0.0**	1.0
**215**	TRINITY_DN35262_c0_g1_i1	ornithine decarboxylase antizyme 2	OAZ2	-4.3	0.2	0.8	**0.0**	0.9	0.8
**216**	TRINITY_DN36464_c1_g1_i6	pancreatic progenitor cell differentiation and proliferation factor-like protein	PPDPFL	5.0	2.2	-1.6	**0.0**	0.2	0.8
**217**	TRINITY_DN29832_c0_g2_i2	PDZ and LIM domain protein 5 isoform X2	PDLIM5	-5.4	0.4	-1.0	**0.0**	0.8	0.7
**218**	TRINITY_DN33680_c0_g2_i5	PDZ and LIM domain protein 7 isoform X4	PDLIM7	-7.4	0.2	-0.4	**0.0**	0.9	0.9
**219**	TRINITY_DN27621_c0_g2_i4	peptidyl-prolyl cis-trans isomerase FKBP3	FKBP3	-3.6	1.5	1.3	**0.0**	0.3	0.6

**220**	TRINITY_DN29857_c1_g1_i1	phosphoglycerate kinase 1	PGK1	-3.3	0.3	-0.2	**0.0**	0.8	0.9
**221**	TRINITY_DN37070_c1_g3_i1	plakophilin-1	PKP1	-1.5	-3.8	-4.4	0.3	**0.0**	0.2
**222**	TRINITY_DN28295_c1_g2_i3	polyubiquitin-B isoform X1	UBB	3.6	2.5	-0.6	**0.0**	0.1	0.9
**223**	TRINITY_DN30180_c0_g1_i2	probable ATP-dependent RNA helicase DDX17	DDX17	-1.2	-3.8	-4.4	0.4	**0.0**	0.2
**224**	TRINITY_DN24163_c0_g2_i1	probable peroxisomal membrane protein PEX13	PEX13	3.6	0.0	0.4	**0.0**	1.0	1.0
**225**	TRINITY_DN35520_c1_g2_i4	proline dehydrogenase 1, mitochondrial-like	PRODH	3.4	1.8	-1.6	**0.0**	0.2	0.6
**226**	TRINITY_DN28789_c0_g1_i4	protein FAM162A	FAM162A	-3.6	0.5	1.6	**0.0**	0.8	0.5
**227**	TRINITY_DN31243_c0_g1_i1	protein FAM184B	FAM184B	3.7	1.6	-0.6	**0.0**	0.6	1.0
**228**	TRINITY_DN36144_c0_g1_i4	protein kinase C and casein kinase substrate in neurons protein 2-like isoform X1	PACSIN2	-3.6	-1.0	-1.2	**0.0**	0.5	0.7
**229**	TRINITY_DN28763_c0_g4_i1	protein SET	SET	-3.2	1.1	1.6	**0.0**	0.4	0.5
**230**	TRINITY_DN37046_c1_g1_i4	pyruvate kinase PKM isoform X1	PKM	-4.0	-0.8	-1.5	**0.0**	0.6	0.6
**231**	TRINITY_DN28890_c1_g2_i9	regulator of cell cycle RGCC-like	RGCC	2.0	3.7	2.2	0.4	**0.0**	0.6
**232**	TRINITY_DN37687_c1_g1_i5	RNA-binding protein 24	RBM24	-3.6	0.2	-0.1	**0.0**	0.9	1.0
**233**	TRINITY_DN38335_c0_g1_i1	ryanodine receptor 1	RYR1	-3.8	-3.6	-5.2	**0.0**	**0.0**	0.1
**234**	TRINITY_DN38019_c1_g3_i2	sarcoplasmic/endoplasmic reticulum calcium ATPase 1	ATP2A1	-4.5	-0.4	-1.1	**0.0**	0.8	0.7
**235**	TRINITY_DN29632_c0_g2_i2	serpin B10-like	SERPINB10	0.9	-6.9	-2.4	0.5	**0.0**	0.4
**236**	TRINITY_DN30685_c0_g2_i5	S-formylglutathione hydrolase	ESD	-3.8	1.1	1.0	**0.0**	0.5	0.7
**237**	TRINITY_DN35169_c0_g3_i1	SH3 domain-binding glutamic acid-rich protein	SH3BGR	-3.5	0.0	-1.7	**0.0**	1.0	0.5
**238**	TRINITY_DN29450_c0_g3_i3	sphingomyelin phosphodiesterase 5	smpd5	1.0	3.9	1.0	1.0	**0.0**	1.0

**239**	TRINITY_DN19422_c0_g1_i2	thioredoxin domain-containing protein 17	TXNDC17	0.0	-0.9	-7.4	1.0	0.5	**0.0**
**240**	TRINITY_DN38339_c2_g2_i1	titin isoform X10	TTN	-1.4	-4.9	-8.3	0.3	**0.0**	**0.0**
**241**	TRINITY_DN25133_c0_g1_i11	trans-1,2-dihydrobenzene-1,2-diol dehydrogenase	DHDH	1.1	-3.8	-5.3	0.4	**0.0**	0.1
**242**	TRINITY_DN37409_c0_g1_i2	transitional endoplasmic reticulum ATPase	VCP	-3.6	-0.1	-0.2	**0.0**	1.0	1.0
**243**	TRINITY_DN37819_c2_g1_i8	translationally-controlled tumor protein	TPT1	-3.2	1.1	1.3	**0.0**	0.5	0.6
**244**	TRINITY_DN28724_c0_g2_i2	transmembrane protein 223	TMEM223	2.3	3.8	2.4	0.2	**0.0**	0.5
**245**	TRINITY_DN37095_c3_g2_i3	triosephosphate isomerase	TPI1	-3.7	-0.6	-0.2	**0.0**	0.7	0.9
**246**	TRINITY_DN37172_c0_g2_i2	tripartite motif-containing protein 72	TRIM72	2.8	1.1	-2.9	**0.0**	0.5	0.4
**247**	TRINITY_DN31007_c0_g2_i5	tropomodulin-4	TMOD4	-3.8	0.7	0.2	**0.0**	0.6	1.0
**248**	TRINITY_DN32390_c3_g1_i2	tropomyosin alpha-3 chain isoform X1	TPM3	-4.5	0.6	0.0	**0.0**	0.7	1.0
**249**	TRINITY_DN38292_c1_g1_i4	troponin I, fast skeletal muscle	TNNI2	-9.3	1.4	0.3	**0.0**	0.3	0.9
**250**	TRINITY_DN28063_c1_g1_i2	troponin I, slow skeletal muscle	TNNI1	-3.5	1.2	0.8	**0.0**	0.4	0.8
**251**	TRINITY_DN24287_c5_g2_i1	troponin T, fast skeletal muscle isoform X1	TNNT3	-5.9	1.0	0.0	**0.0**	0.4	1.0
**252**	TRINITY_DN27210_c1_g2_i1	ubiquitin-60S ribosomal protein L40	UBA52	-4.7	1.2	1.7	**0.0**	0.4	0.5
**253**	TRINITY_DN30472_c0_g1_i3	vitamin D3 hydroxylase-associated protein-like	VDHAP	-3.2	1.0	0.2	**0.0**	0.5	0.9
**254**	TRINITY_DN38095_c1_g1_i2	zinc finger protein 106 isoform X1	ZNF106	-1.2	-4.0	-4.6	0.4	**0.0**	0.2

Supplementary Table 2: List of genes involved in various GO biological functions. Biological Functions, No of genes involved in the biological function, p-value and The list of genes involved in the biological functions were shown in each column.

Functional Category	No. of Genes	p-Value	Genes List
Unclassified (UNCLASSIFIED)	13	0.00019	MYH7B ACTN3 KRT23 BZW1 RPS25 MYL6 RBP5 FAM184B SGCA TMEM128 MYOZ3 PVALB SH3BGR
cellular component organization (GO:0016043)	50	0.00020	TRIM72 ATF2 ATP2A1 POF1B MYOZ1 MAP3K7 FGB DSP POSTN ANXA2 TPM3 TMOD4 TPM1 ATG9A KRT1 ELF3 BIN1 MYL2 MYOD1 TNNT3 LUC7L3 Lnpk PKP1 CASQ1 VCP EPB41 DOT1L SNU13 NDRG1 DEDD2 PSAP PACSIN2 XIRP2 SFN PDLIM5 PDLIM7 RNF20 HSPA8 SLC16A1 ADRM1 NEB PEX13 COL1A1 AFDN MYO1B KRT19 COL3A1 DES ACTC1 VAPB
cellular component organization or biogenesis (GO:0071840)	53	0.00007	TRIM72 ATF2 ATP2A1 POF1B MYOZ1 MAP3K7 FGB DSP POSTN ANXA2 TPM3 RPS24 RPL35A GLUL TMOD4 TPM1 ATG9A KRT1 ELF3 BIN1 MYL2 MYOD1 TNNT3 LUC7L3 Lnpk PKP1 CASQ1 VCP EPB41 DOT1L SNU13 NDRG1 DEDD2 PSAP PACSIN2 XIRP2 SFN PDLIM5 PDLIM7 RNF20 HSPA8 SLC16A1 ADRM1 NEB PEX13 COL1A1 AFDN MYO1B KRT19 COL3A1 DES ACTC1 VAPB
multicellular organismal process (GO:0032501)	47	0.00019	MYOM1 ATF2 DLX3 CEBPD ITGB2 HOXD13 ATP2A1 NDRG1 PSAP TNNI1 PPDPFL XIRP2 TNNI2 SFN PDLIM5 PAIP2 NDUFV2 MYOZ1 PDLIM7 FGB DSP MYBPC2 ANXA2 TPM3 PLLP TMOD4 TPM1 NEB PEX13 GRIP2 COL1A1 AFDN COL3A1 RGCC ACTC1 ASPH BIN1 MYL1 MYL2 MYOD1 TNNT3 angptl7 ADAM9 Lnpk PPARA RBM24 TPT1
developmental process (GO:0032502)	44	0.00009	CD63 ATF2 DLX3 CEBPD ITGB2 HOXD13 NDRG1 POF1B PSAP PACSIN2 TNNI1 PPDPFL XIRP2 SFN PDLIM5 PAIP2 NDUFV2 MYOZ1 HRAS PDLIM7 DSP POSTN ANXA2 PLLP TMOD4 NEB PEX13 COL1A1 AFDN KRT19 COL3A1 ACTC1 ASPH ELF3 BIN1 MYL2 MYOD1 TNNT3 angptl7 ADAM9 Lnpk PPARA RBM24 TPT1
response to stress (GO:0006950)	31	0.00002	TRIM72 ATF2 VCP DOT1L ATP2A1 NDRG1 HERPUD1 FAM162A UCP3 PCLAF SFN MYOZ1 MAP3K7 HRAS FGB DSP HSPA8 KRT1 COL3A1 RGCC ELF3 VAPB MYOD1 ADAM9 Lnpk TMBIM6 P4HB PPARA GAPDH RBM24 FAN1
anatomical structure morphogenesis (GO:0009653)	27	0.00002	ATF2 DLX3 ITGB2 HOXD13 POF1B PSAP PACSIN2 TNNI1 XIRP2 MYOZ1 DSP ANXA2 TMOD4 NEB COL1A1 AFDN KRT19 COL3A1 ACTC1 ASPH ELF3 MYL2 MYOD1 TNNT3 angptl7 Lnpk PPARA
cellular response to stress (GO:0033554)	20	0.00013	ATF2 HSPA8 VCP DOT1L ATP2A1 NDRG1 HERPUD1 RGCC FAM162A VAPB MYOD1 PCLAF SFN TMBIM6 P4HB PPARA MAP3K7 HRAS RBM24 FAN1
regulation of hydrolase	17	0.00028	SCARB2 SERPINB10 VCP ANXA2 RARRES1 ADRM1 SPINT1 ASPH FAM162A BIN1 TNNT3 PSAP SFN TMBIM6 MYOZ1 GAPDH

activity (GO:0051336)			HRAS
system process (GO:0003008)	19	0.00011	FGB MYOM1 MYBPC2 TPM3 TMOD4 TPM1 ATP2A1 GRIP2 COL1A1 ACTC1 MYL1 MYL2 MYOD1 TNNT3 PSAP TNNI1 TNNI2 PAIP2 MYOZ1
cellular nitrogen compound biosynthetic process (GO:0044271)	20	0.00003	EIF4A2 VCP ATP5PD ADRM1 ATP5MC2 MRPL17 RPL35A ATP5MC1 EEF1B2 MTAP HINT1 ELF3 IMPDH1 PCLAF ADSSL1 RPL18 RPS11 UBA52 RPS24 RPS12
cytoskeleton organization (GO:0007010)	21	0.00001	DSP VCP SLC16A1 TPM3 EPB41 TMOD4 TPM1 NEB MYO1B KRT19 DES ACTC1 POF1B MYL2 TNNT3 PACSIN2 XIRP2 PKP1 PDLIM5 MYOZ1 PDLIM7
regulation of protein localization (GO:0032880)	13	0.00028	FGB MYOM1 SLC16A1 EPB41 OAZ2 AFDN RGCC ADAM9 SFN TMBIM6 GAPDH HRAS GLUL
tissue development (GO:0009888)	24	0.00000	DSP ATF2 POSTN DLX3 ITGB2 NEB HXD13 COL1A1 AFDN COL3A1 ACTC1 POF1B ELF3 MYL2 MYOD1 PSAP TNNI1 ADAM9 XIRP2 SFN PPARA NDUFV2 MYOZ1 RBM24
positive regulation of transport (GO:0051050)	12	0.00024	FGB CD63 MYOM1 RGCC ANXA2 ITGB2 ADAM9 SFN CASQ1 GAPDH HRAS OAZ2
anatomical structure formation involved in morphogenesis (GO:0048646)	14	0.00006	ANXA2 TMOD4 ITGB2 NEB COL1A1 KRT19 ACTC1 MYL2 MYOD1 TNNT3 PSAP angptl7 PPARA MYOZ1
regulation of establishment of protein localization (GO:0070201)	10	0.00030	FGB MYOM1 RGCC SLC16A1 ADAM9 SFN TMBIM6 GAPDH HRAS OAZ2
circulatory system development (GO:0072359)	16	0.00000	DSP ATF2 DLX3 ANXA2 NEB COL1A1 COL3A1 ACTC1 MYL2 TNNI1 angptl7 XIRP2 PDLIM5 NDUFV2 RBM24 PDLIM7
regulation of peptide transport (GO:0090087)	10	0.00023	FGB MYOM1 RGCC SLC16A1 ADAM9 SFN TMBIM6 GAPDH HRAS OAZ2
nucleobase-containing small molecule	12	0.00004	HINT1 VCP PKM TPI1 ATP5PD IMPDH1 PGK1 ADSSL1 ATP5MC2 GAPDH ATP5MC1 MTAP

metabolic process (GO:0055086)			
regulation of protein transport (GO:0051223)	10	0.00018	FGB MYOM1 RGCC SLC16A1 ADAM9 SFN TMBIM6 GAPDH HRAS OAZ2
negative regulation of hydrolase activity (GO:0051346)	10	0.00017	SERPINB10 SPINT1 ANXA2 BIN1 RARRES1 SFN TMBIM6 MYOZ1 GAPDH HRAS
regulation of system process (GO:0044057)	9	0.00027	FGB DSP BIN1 MYL2 TNNT3 TNNI1 ATP2A1 CASQ1 CAMK2G
actin filament-based process (GO:0030029)	16	0.00000	TPM3 EPB41 TMOD4 TPM1 NEB MYO1B KRT19 ACTC1 POF1B MYL2 TNNT3 PACSIN2 XIRP2 PDLIM5 MYOZ1 PDLIM7
nucleoside phosphate metabolic process (GO:0006753)	11	0.00004	HINT1 VCP PKM TPI1 ATP5PD IMPDH1 PGK1 ADSSL1 ATP5MC2 GAPDH ATP5MC1
regulation of peptidase activity (GO:0052547)	11	0.00003	SERPINB10 VCP SPINT1 ASPH ANXA2 FAM162A BIN1 RARRES1 ADRM1 SFN GAPDH
actin cytoskeleton organization (GO:0030036)	16	0.00000	TPM3 EPB41 TMOD4 TPM1 NEB MYO1B KRT19 ACTC1 POF1B MYL2 TNNT3 PACSIN2 XIRP2 PDLIM5 MYOZ1 PDLIM7
nucleotide metabolic process (GO:0009117)	11	0.00002	HINT1 VCP PKM TPI1 ATP5PD IMPDH1 PGK1 ADSSL1 ATP5MC2 GAPDH ATP5MC1
heart development (GO:0007507)	12	0.00001	DSP ATF2 COL3A1 ACTC1 MYL2 TNNI1 XIRP2 NEB PDLIM5 NDUFV2 RBM24 PDLIM7
regulation of endopeptidase activity (GO:0052548)	11	0.00001	SERPINB10 VCP SPINT1 ASPH ANXA2 FAM162A BIN1 RARRES1 ADRM1 SFN GAPDH
ribose phosphate metabolic process (GO:0019693)	10	0.00002	HINT1 PKM TPI1 ATP5PD IMPDH1 PGK1 ADSSL1 ATP5MC2 GAPDH ATP5MC1

purine-containing compound metabolic process (GO:0072521)	11	0.00001	HINT1 MTAP PKM TPI1 ATP5PD IMPDH1 PGK1 ADSSL1 ATP5MC2 GAPDH ATP5MC1
supramolecular fiber organization (GO:0097435)	17	0.00000	DSP ANXA2 TPM3 TMOD4 TPM1 NEB COL1A1 MYO1B KRT19 COL3A1 DES ACTC1 POF1B MYL2 TNNT3 PKP1 MYOZ1
regulation of protein secretion (GO:0050708)	7	0.00030	FGB MYOM1 RGCC SLC16A1 ADAM9 TMBIM6 GAPDH
purine nucleotide metabolic process (GO:0006163)	10	0.00001	HINT1 PKM TPI1 ATP5PD IMPDH1 PGK1 ADSSL1 ATP5MC2 GAPDH ATP5MC1
ribonucleotide metabolic process (GO:0009259)	10	0.00001	HINT1 PKM TPI1 ATP5PD IMPDH1 PGK1 ADSSL1 ATP5MC2 GAPDH ATP5MC1
positive regulation of establishment of protein localization (GO:1904951)	8	0.00008	FGB MYOM1 RGCC ADAM9 SFN GAPDH HRAS OAZ2
striated muscle tissue development (GO:0014706)	9	0.00003	DSP ACTC1 MYL2 MYOD1 TNNI1 XIRP2 NEB NDUFV2 MYOZ1
purine ribonucleotide metabolic process (GO:0009150)	10	0.00001	HINT1 PKM TPI1 ATP5PD IMPDH1 PGK1 ADSSL1 ATP5MC2 GAPDH ATP5MC1
muscle tissue development (GO:0060537)	10	0.00001	DSP COL3A1 ACTC1 MYL2 MYOD1 TNNI1 XIRP2 NEB NDUFV2 MYOZ1
wound healing (GO:0042060)	7	0.00017	FGB DSP TRIM72 COL3A1 Lnpk PPARA MYOZ1
positive regulation of protein transport	8	0.00004	FGB MYOM1 RGCC ADAM9 SFN GAPDH HRAS OAZ2

(GO:0051222)			
muscle organ development (GO:0007517)	8	0.00004	DSP COL3A1 ACTC1 MYL2 TNNI1 XIRP2 MYOD1 MYOZ1
muscle structure development (GO:0061061)	15	0.00000	DSP TMOD4 NEB KRT19 COL3A1 ACTC1 BIN1 MYL2 MYOD1 TNNT3 TNNI1 XIRP2 PDLIM5 MYOZ1 PDLIM7
cell-substrate adhesion (GO:0031589)	7	0.00009	FGB CD63 COL3A1 ITGB2 ADAM9 LYPD3 FERMT2
muscle cell differentiation (GO:0042692)	9	0.00001	KRT19 ACTC1 TMOD4 MYL2 MYOD1 TNNT3 NEB MYOZ1 BIN1
regulation of RNA splicing (GO:0043484)	7	0.00007	HSPA8 HNRNPH1 MYOD1 CIRBP SRSF2 TMBIM6 RBM24
proton transmembrane transport (GO:1902600)	6	0.00022	ATP5PD UCP3 ATP5MC2 ND5 ATP5MC1
striated muscle cell differentiation (GO:0051146)	8	0.00002	KRT19 ACTC1 TMOD4 MYL2 MYOD1 TNNT3 NEB MYOZ1
regulation of muscle system process (GO:0090257)	7	0.00003	DSP BIN1 TNNT3 TNNI1 ATP2A1 CASQ1 CAMK2G
cardiac muscle tissue development (GO:0048738)	7	0.00002	DSP ACTC1 MYL2 TNNI1 XIRP2 NEB NDUFV2
carbohydrate catabolic process (GO:0016052)	5	0.00023	PKM TPI1 PGK1 PYGM GAPDH
heart process (GO:0003015)	5	0.00015	ACTC1 MYL2 TPM1 TNNI1 TNNI2
cellular response to oxygen levels (GO:0071453)	5	0.00015	RGCC FAM162A MYOD1 P4HB NDRG1
cell-matrix adhesion (GO:0007160)	7	0.00001	FGB CD63 COL3A1 ITGB2 ADAM9 LYPD3 FERMT2
striated muscle	8	0.00000	KRT19 ACTC1 TMOD4 MYL2 MYOD1 TNNT3 NEB MYOZ1

cell development (GO:0055002)			
actomyosin structure organization (GO:0031032)	8	0.00000	KRT19 ACTC1 TMOD4 MYL2 MYOD1 TNNT3 NEB MYOZ1
striated muscle contraction (GO:0006941)	5	0.00011	MYL2 TNNT3 TPM1 TNNI1 TNNI2
skin development (GO:0043588)	6	0.00002	DSP PSAP ADAM9 SFN COL1A1 COL3A1
ATP metabolic process (GO:0046034)	11	0.00000	HSPA8 VCP PKM TPI1 ATP5PD PGK1 ATP5MC2 NDUFV2 GAPDH ND5 ATP5MC1
muscle cell development (GO:0055001)	9	0.00000	KRT19 ACTC1 TMOD4 MYL2 MYOD1 TNNT3 NEB MYOZ1 BIN1
muscle contraction (GO:0006936)	11	0.00000	MYOM1 MYBPC2 TPM3 TMOD4 MYL1 MYL2 TNNT3 TPM1 TNNI1 TNNI2 GRIP2
heart contraction (GO:0060047)	5	0.00008	ACTC1 MYL2 TPM1 TNNI1 TNNI2
regulation of striated muscle contraction (GO:0006942)	5	0.00008	DSP BIN1 TNNT3 ATP2A1 CASQ1
cellular component assembly involved in morphogenesis (GO:0010927)	7	0.00000	KRT19 ACTC1 TMOD4 MYL2 TNNT3 NEB MYOZ1
muscle system process (GO:0003012)	14	0.00000	MYOM1 MYBPC2 TPM3 TMOD4 TPM1 ATP2A1 GRIP2 MYL1 MYL2 MYOD1 TNNT3 TNNI1 TNNI2 MYOZ1
ribonucleoside triphosphate biosynthetic process (GO:0009201)	4	0.00029	ATP5PD IMPDH1 ATP5MC2 ATP5MC1
purine ribonucleoside diphosphate metabolic	4	0.00029	PKM TPI1 PGK1 GAPDH

process (GO:0009179)			
purine nucleoside diphosphate metabolic process (GO:0009135)	4	0.00029	PKM TPI1 PGK1 GAPDH
muscle organ morphogenesis (GO:0048644)	6	0.00000	DSP COL3A1 ACTC1 MYL2 TNNI1 XIRP2
ADP metabolic process (GO:0046031)	4	0.00019	PKM TPI1 PGK1 PYGM GAPDH
purine nucleoside triphosphate biosynthetic process (GO:0009145)	4	0.00017	ATP5PD IMPDH1 ATP5MC2 ATP5MC1
cardiac muscle tissue morphogenesis (GO:0055008)	5	0.00002	DSP ACTC1 MYL2 TNNI1 XIRP2
purine ribonucleoside triphosphate biosynthetic process (GO:0009206)	4	0.00016	ATP5PD IMPDH1 ATP5MC2 ATP5MC1
muscle tissue morphogenesis (GO:0060415)	6	0.00000	DSP COL3A1 ACTC1 MYL2 TNNI1 XIRP2
ATP generation from ADP (GO:0006757)	4	0.00014	PKM TPI1 PGK1 PYGM GAPDH
glycolytic process (GO:0006096)	4	0.00012	PKM TPI1 PGK1 GAPDH
keratinocyte differentiation (GO:0030216)	4	0.00012	DSP PSAP ADAM9 SFN
myofibril assembly (GO:0030239)	7	0.00000	KRT19 ACTC1 TMOD4 MYL2 TNNT3 NEB MYOZ1

Supplementary Table 3: List of differentially expressed protein based on iTRAQ proteomic analysis. Accession, description, symbol, No of peptides identified, peptide sequence matches, 1dpa fold changes, 2dpa fold changes, 5dpa fold changes, 1dpa SEM, 2dpa SEM and 5dpa SEM were shown in each column.

S.No	Accession	Description	Symbol	# Peptides	# PSMs	Protein fold change			SEM		
						1-dpa	2-dpa	5-dpa	1-dpa	2-dpa	5-dpa
1	TRINITY_DN23220_c0_g2_i4	hemoglobin subunit beta	HBB	3	3	3.85	2.79	-0.85	0.29	0.68	0.59
2	TRINITY_DN34721_c0_g1_i1	myosin-binding protein H	MYBPH	1	1	3.17	0.32	-1.82	0.13	0.13	0.19
3	TRINITY_DN19815_c3_g1_i2	hemoglobin subunit alpha-D	HBAD	2	4	3.13	1.66	-1.74	0.66	0.76	0.50
4	TRINITY_DN31561_c0_g6_i1	S100-A7-like	S100A7	3	5	2.86	0.29	-0.66	0.21	2.34	4.06
5	TRINITY_DN23557_c1_g5_i3	TRINITY_DN23557_c1_g5_i3	N/A	1	1	2.85	-1.45	-3.66	0.16	0.22	0.33
6	TRINITY_DN22863_c0_g2_i2	histone H2A.Z	H2AFZ	4	8	2.68	1.34	-0.39	1.08	0.73	0.15
7	TRINITY_DN36348_c1_g1_i1	Annexin A2	ANXA2	4	7	2.20	1.06	-0.37	0.46	0.34	0.54
8	TRINITY_DN33300_c0_g3_i5	TRINITY_DN33300_c0_g3_i5	N/A	8	12	2.19	1.36	-0.48	0.05	0.21	0.25
9	TRINITY_DN36487_c0_g2_i1	cathepsin B	CTSB	1	2	2.05	0.07	-1.83	0.18	0.06	**0.03**
10	TRINITY_DN34850_c1_g2_i1	mimecan	OGN	4	6	2.03	-0.06	-2.48	0.06	0.32	0.73
11	TRINITY_DN29980_c3_g2_i1	myosin light chain 1/3, skeletal muscle isoform isoform X2	MYL1	6	10	1.95	1.51	-0.97	0.56	0.40	0.41
12	TRINITY_DN23998_c0_g1_i3	tubulin beta chain	TUBB	5	6	1.80	0.89	-1.08	0.20	0.24	**0.03**
13	TRINITY_DN23258_c0_g1_i1	histone H2B 5	H2B-V	2	4	1.79	-0.06	-1.59	0.74	0.81	0.82
14	TRINITY_DN36141_c1_g1_i2	L-lactate dehydrogenase A chain	LDHA	8	10	1.76	1.09	-2.44	0.73	0.63	2.83
15	TRINITY_DN26001_c2_g1_i2	alpha-actinin-1 isoform X1	ACTN1	2	2	1.70	1.13	-0.68	0.37	0.45	0.29
16	TRINITY_DN25724_c1_g3_i3	vimentin	VIM	6	6	1.68	1.00	-0.97	0.61	0.43	0.38
17	TRINITY_DN38128_c1_g2_i3	myosin-binding protein C, slow-type isoform X6	MYBPC1	7	13	1.64	0.99	-0.55	2.45	0.45	0.77
18	TRINITY_DN12736_c0_g1_i1	apolipoprotein A-I	APOA1	5	10	1.64	-0.63	-2.25	0.20	0.50	0.73
19	TRINITY_DN36902_c2_g1_i2	alcohol dehydrogenase 1	Adh1	1	1	1.63	1.74	-1.76	0.05	**0.02**	**0.04**
20	TRINITY_DN26228_c0_g1_i7	short chain dehydrogenase gsfK-like	GSFK	1	1	1.63	-2.24	-5.82	**0.03**	0.25	0.34

21	TRINITY_DN36364_c0_g1_i7	tubulin alpha-1A chain	TUBA1A	4	4	1.62	0.86	-1.22	0.72	0.71	0.64
22	TRINITY_DN37921_c1_g2_i2	keratin, type I cytoskeletal 24-like	KRT24	8	8	1.6	1.4	-0.5	0.61	0.58	0.07
23	TRINITY_DN34661_c0_g1_i3	phosphoglucomutase-1 isoform X2	PGM1	7	11	1.55	1.58	-1.01	0.46	0.23	0.07
24	TRINITY_DN27002_c0_g1_i1	actin, cytoplasmic 2	ACTG1	21	48	1.52	1.19	-0.44	0.69	0.53	0.30
25	TRINITY_DN32459_c0_g1_i1	calpain small subunit 1	CAPNS1	1	1	1.52	-0.46	-4.13	0.08	**0.03**	1.27
26	TRINITY_DN21930_c0_g2_i3	Proteasome subunit beta type-2	PSMB2	1	1	1.45	-0.72	-2.97	0.11	**0.02**	0.14
27	TRINITY_DN20734_c0_g4_i1	Actin alpha cardiac muscle 1	ACTC1	23	65	1.41	1.57	-1.00	0.53	0.37	0.36
28	TRINITY_DN34313_c0_g1_i4	ATP synthase subunit alpha, mitochondrial	ATP1	2	2	1.35	0.53	-1.18	**0.02**	**0.04**	0.09
29	TRINITY_DN29508_c0_g1_i2	TRINITY_DN29508_c0_g1_i2	N/A	1	1	1.31	0.91	-1.34	0.10	0.06	0.08
30	TRINITY_DN37043_c2_g2_i2	creatine kinase M-type	CKM	9	16	1.28	1.04	-1.61	0.60	0.58	0.42
31	TRINITY_DN27922_c3_g1_i4	adenylate kinase isoenzyme 1 isoform X1	AK1	3	6	1.26	-0.39	-1.29	1.00	0.73	0.42
32	TRINITY_DN33300_c0_g3_i4	TRINITY_DN33300_c0_g3_i4	N/A	13	22	1.26	0.56	-1.68	0.54	0.56	0.72
33	TRINITY_DN36070_c0_g2_i4	LIM domain-binding protein 3 isoform X12	LDB3	2	2	1.15	0.30	-1.17	0.25	0.14	0.40
34	TRINITY_DN28295_c1_g2_i4	polyubiquitin-C	UBC	1	1	1.14	2.13	-0.29	0.84	0.76	0.15
35	TRINITY_DN37255_c1_g2_i2	leukotriene A-4 hydrolase	LKA4	1	1	1.13	1.51	0.23	0.22	0.25	0.11
36	TRINITY_DN38343_c2_g2_i1	myosin-1B-like isoform X5	MYO1B	35	60	1.08	1.45	-1.12	1.15	0.31	0.56
37	TRINITY_DN34296_c3_g8_i1	actin, alpha cardiac muscle 1	ACTC1	15	43	1.07	1.23	-0.58	0.36	0.42	0.41
38	TRINITY_DN23035_c0_g2_i2	dihydrolipoyl dehydrogenase, mitochondrial	DLD	3	3	1.1	0.5	-0.7	0.98	0.76	0.06
39	TRINITY_DN33310_c0_g1_i2	histone H2A type 2-B-like	HIST2H2AB	1	1	1.07	-0.88	-1.71	1.09	0.84	0.50
40	TRINITY_DN38292_c1_g1_i4	troponin I, fast skeletal muscle	TNNI2	4	5	1.06	0.77	-0.96	1.27	0.42	0.50
41	TRINITY_DN32404_c3_g1_i6	actin aortic smooth muscle	ACTA2	19	53	1.06	0.61	-1.32	0.62	0.28	0.33
42	TRINITY_DN30191_c0_g7_i1	F-actin-capping protein subunit beta isoform X2	CAPZB	1	1	1.00	-0.30	-3.88	**0.05**	0.06	0.14
43	TRINITY_DN28501_c1_g1_i4	synaptic vesicle membrane protein VAT-1 homolog	VAT1	1	1	0.99	0.32	-1.00	**0.03**	0.10	**0.04**
44	TRINITY_DN28891_c0_g1_i2	malate dehydrogenase, mitochondrial	MDH2	2	2	0.98	0.00	-2.67	0.06	0.11	0.36
45	TRINITY_DN32432_c1_g2_i2	myozenin-1 isoform X1	MYOZ1	4	5	1.0	1.1	-1.2	0.6	0.6	0.3

46	TRINITY_DN28342_c0_g1_i2	myosin heavy chain, skeletal muscle-like	MYH	9	13	0.97	1.56	-0.36	1.05	0.42	0.45
47	TRINITY_DN27358_c0_g1_i3	glutathione S-transferase-like	GST	2	2	0.92	-1.65	-4.33	0.14	0.31	**0.03**
48	TRINITY_DN27718_c1_g2_i1	desmin	DES	4	5	0.92	0.42	-0.87	0.44	0.23	0.13
49	TRINITY_DN36557_c1_g1_i2	fructose-bisphosphate aldolase C	ALDOC	5	7	0.86	0.88	-1.57	0.62	0.29	1.06
50	TRINITY_DN22922_c0_g1_i4	galectin-3 isoform X2	LGALS3	1	1	0.81	-0.34	-2.48	**0.00**	0.08	0.09
51	TRINITY_DN34182_c1_g1_i1	TRINITY_DN34182_c1_g1_i1	N/A	20	68	0.80	0.93	-0.54	0.63	0.42	0.28
52	TRINITY_DN22784_c0_g1_i2	TRINITY_DN22784_c0_g1_i2	N/A	13	20	0.79	0.15	-3.16	0.55	0.37	0.81
53	TRINITY_DN29857_c1_g1_i1	phosphoglycerate kinase 1	PGK1	3	4	0.8	0.8	-1.0	**0.03**	0.07	**0.02**
54	TRINITY_DN37459_c2_g1_i2	alpha-actinin-2 isoform X1	LOC100179322	16	17	0.77	1.79	-0.15	0.88	0.75	0.72
55	TRINITY_DN37078_c1_g1_i8	cytosol aminopeptidase	LAP3	1	1	0.69	1.59	0.63	0.34	0.44	0.21
56	TRINITY_DN33047_c0_g1_i1	heat shock protein HSP 90-alpha	HSP90AA1	2	2	0.68	0.86	-0.62	0.94	**0.04**	**0.01**
57	TRINITY_DN37032_c0_g2_i1	heat shock cognate 71 kDa protein	HSPA8	1	1	0.67	0.85	-0.92	0.93	0.57	0.45
58	TRINITY_DN34564_c0_g1_i6	annexin A6 isoform X1	ANXA6	1	1	0.64	0.80	-0.93	0.81	0.77	0.25
59	TRINITY_DN28342_c0_g2_i2	heat shock cognate 71 kDa protein-like	HSPA8	2	2	0.61	0.90	-0.54	0.82	0.42	0.12
60	TRINITY_DN38304_c0_g1_i4	TRINITY_DN38304_c0_g1_i4	N/A	1	1	0.60	-0.22	-2.08	1.16	0.06	1.17
61	TRINITY_DN29028_c0_g2_i3	phosphoglycerate mutase 1	PGAM1	6	8	0.58	1.12	0.31	0.54	1.05	0.23
62	TRINITY_DN27789_c2_g1_i2	phosphoglycerate mutase 2	PGAM2	5	12	0.54	-0.16	-3.49	**0.02**	0.10	0.06
63	TRINITY_DN26466_c0_g2_i5	Protein O-GlcNAcase	OGA	10	16	0.50	0.64	0.12	0.39	0.32	0.48
64	TRINITY_DN36562_c0_g1_i9	glutathione S-transferase Mu 1-like	GSTM1	2	4	0.49	-0.01	-1.11	0.31	0.12	0.55
65	TRINITY_DN31350_c0_g1_i3	peroxiredoxin-6	PRDX6	1	1	0.49	-0.63	-3.76	**0.03**	**0.01**	0.28
66	TRINITY_DN28781_c0_g1_i3	pyruvate kinase PKM isoform X2	PKM	1	1	0.47	0.77	-0.55	1.45	0.86	0.28
67	TRINITY_DN37517_c6_g2_i1	parvalbumin beta	PVALB	3	3	0.46	1.66	-1.51	0.31	1.30	0.51
68	TRINITY_DN26606_c2_g2_i1	F-actin-capping protein subunit alpha-2	CAPZA2	1	1	0.45	-0.39	-3.42	0.07	0.08	**0.01**
69	TRINITY_DN37046_c1_g1_i4	pyruvate kinase PKM isoform X1	PKM	6	6	0.45	1.01	-0.96	0.58	0.33	0.39
70	TRINITY_DN33370_c2_g2_i3	keratin, type II cytoskeletal 5-like	KRT5	7	8	0.4	0.8	-0.6	0.15	0.31	0.29

71	TRINITY_DN34011_c0_g1_i3	myosin-1B-like isoform X2	MYO1B	2	3	0.44	0.83	-4.91	**0.00**	**0.01**	0.05
72	TRINITY_DN29832_c0_g2_i2	PDZ and LIM domain protein 5 isoform X2	PDLIM5	2	3	0.39	-0.64	-3.40	0.14	0.14	0.20
73	TRINITY_DN37554_c1_g1_i2	glycogen phosphorylase, muscle form	PYGM	16	27	0.38	0.70	-1.66	0.50	0.34	0.45
74	TRINITY_DN22345_c2_g1_i1	Parvalbumin alpha	PVALB	2	3	0.37	1.78	-0.06	1.18	0.55	0.97
75	TRINITY_DN29856_c0_g1_i2	carbonyl reductase [NADPH] 1-like	CBR1	1	1	0.33	-0.65	-3.32	**0.04**	0.09	0.15
76	TRINITY_DN34096_c0_g1_i3	aldose reductase-like	AR	2	3	0.33	-0.49	-3.31	**0.03**	**0.03**	0.27
77	TRINITY_DN38029_c0_g1_i4	collagen alpha-1(I) chain	COL1A1	26	30	0.28	-0.18	-0.45	0.28	0.81	1.02
78	TRINITY_DN37645_c3_g2_i1	myosin regulatory light chain 2, skeletal muscle isoform	MYLPF	2	4	0.26	2.91	-0.65	**0.05**	0.07	0.10
79	TRINITY_DN22413_c0_g2_i1	N-acetylneuraminate lyase	NPL	3	6	0.18	1.11	0.86	0.30	0.92	0.91
80	TRINITY_DN32810_c4_g1_i4	fructose-bisphosphate aldolase A	ALDOA	8	35	0.18	1.34	-0.71	0.41	0.42	0.52
81	TRINITY_DN32365_c1_g2_i2	myozenin-3	MYOZ3	1	1	0.11	-0.55	-3.38	0.07	0.10	0.27
82	TRINITY_DN37786_c0_g1_i2	collagen alpha-1(VI) chain	COL6A1	5	5	0.04	0.21	-1.95	0.22	0.47	1.20
83	TRINITY_DN34741_c2_g2_i1	heat shock protein HSP 90-beta	HSP90AB1	2	2	0.03	0.81	-1.10	0.11	0.08	0.08
84	TRINITY_DN26737_c2_g1_i1	myosin-3-like isoform X7	MYH3	12	17	-0.01	1.09	-0.84	0.74	0.17	0.62
85	TRINITY_DN33401_c0_g2_i2	complement component 1 Q subcomponent-binding protein, mitochondrial	C1QBP	1	1	-0.02	-0.30	-2.58	**0.03**	**0.02**	0.17
86	TRINITY_DN29973_c3_g1_i6	troponin T, fast skeletal muscle isoform X1	TNNT3	3	10	-0.08	-0.24	-3.64	0.35	0.07	0.34
87	TRINITY_DN37310_c1_g1_i4	plectin isoform X4	PLEC1	1	1	-0.08	0.45	-0.76	**0.00**	**0.04**	**0.04**
88	TRINITY_DN37095_c3_g2_i3	triosephosphate isomerase	TPI1	8	14	-0.09	-0.34	-5.37	1.20	0.52	0.70
89	TRINITY_DN29661_c3_g1_i3	tropomyosin alpha-3 chain isoform X1	TPM3	7	8	-0.17	-0.11	-4.27	0.19	**0.03**	0.06
90	TRINITY_DN35037_c0_g1_i3	glucose-6-phosphate isomerase	GPI	3	3	-0.18	0.54	-0.90	0.39	0.12	0.05
91	TRINITY_DN37574_c0_g1_i1	ubiquitin-like modifier-activating enzyme 1	UBA1	1	1	-0.18	1.09	-1.11	**0.03**	0.07	0.07
92	TRINITY_DN35510_c0_g1_i3	myelin basic protein isoform X2	MBP	2	3	-0.21	0.29	-1.88	0.86	0.65	0.86
93	TRINITY_DN32170_c0_g3_i2	glycerol-3-phosphate dehydrogenase [NAD(+)], cytoplasmic	GPD1	2	2	-0.23	-0.37	-3.79	0.62	0.38	**0.01**

94	TRINITY_DN37021_c1_g1_i5	alpha-enolase isoform X1	ENO1	12	17	-0.23	1.01	-1.30	0.75	0.60	0.32
95	TRINITY_DN16558_c0_g2_i1	collagen alpha-3(VI) chain isoform X10	COL6A3	1	1	-0.23	0.37	-2.25	0.44	0.44	0.43
96	TRINITY_DN37508_c1_g1_i1	collagen alpha-2(I) chain	COL1A2	14	18	-0.27	-0.43	-2.40	0.54	0.84	0.08
97	TRINITY_DN28227_c0_g1_i2	ADP/ATP translocase 3	SLC25A6	1	1	-0.32	0.49	-1.24	**0.03**	**0.00**	0.07
98	TRINITY_DN37706_c0_g3_i4	periostin isoform X1	POSTN	3	3	-0.35	0.77	-1.38	**0.04**	**0.03**	0.31
99	TRINITY_DN36097_c0_g1_i2	nebulin	NEB	2	2	-0.40	0.45	-1.22	**0.03**	**0.02**	0.28
100	TRINITY_DN26034_c1_g1_i1	transitional endoplasmic reticulum ATPase-like	VCP	1	1	-0.42	0.80	-0.85	0.14	0.08	0.20
101	TRINITY_DN18038_c1_g2_i1	collagen alpha-1(XV) chain isoform X1	COL15A1	1	2	-0.44	0.65	-1.92	0.14	**0.03**	0.10
102	TRINITY_DN37512_c1_g1_i6_	hydroperoxide isomerase ALOXE3-like	ALOXE3	2	4	-0.5	0.2	-1.7	0.28	0.30	0.08
103	TRINITY_DN35151_c1_g3_i3	desmocollin-1 isoform X1	DSC1	1	5	-0.54	0.28	-0.72	0.22	0.20	0.36
104	TRINITY_DN18988_c0_g3_i1	titin-like isoform X23	TTN	2	2	-0.55	0.72	-1.28	0.19	0.16	0.12
105	TRINITY_DN37493_c2_g1_i1	myosin-7B isoform X2	MYH7B	16	30	-0.61	0.60	-0.50	0.09	**0.02**	0.71
106	TRINITY_DN38339_c2_g3_i1	titin isoform X19	TTN	9	11	-0.6	0.8	-1.6	**0.05**	0.13	0.23
107	TRINITY_DN38338_c2_g1_i4	myosin-7-like	MYH7	6	7	-0.62	-0.10	1.22	0.07	0.62	0.25
108	TRINITY_DN38296_c2_g1_i3_	ryanodine receptor 1	RYR1	1	1	-0.6	0.8	-1.0	**0.01**	0.05	0.32
109	TRINITY_DN38142_c2_g2_i1	myomesin-2	MYOM1	6	7	-0.66	0.35	-1.61	**0.02**	0.07	0.08
110	TRINITY_DN37478_c0_g1_i2	glyceraldehyde-3-phosphate dehydrogenase 2	GAPDHS	8	8	-0.67	0.03	-3.11	0.57	0.30	0.90
111	TRINITY_DN19098_c0_g1_i1	collagen alpha-2(VI) chain isoform X1	COL6A2	2	3	-0.67	0.72	-0.73	**0.03**	0.11	**0.03**
112	TRINITY_DN38157_c0_g1_i1	myomesin-1 isoform X2	MYOM1	2	2	-0.72	0.62	-1.58	**0.02**	**0.00**	**0.00**
113	TRINITY_DN36747_c0_g1_i5	AMP deaminase 1	AMPD1	2	2	-0.78	0.46	-0.70	**0.01**	**0.01**	0.11
114	TRINITY_DN38132_c4_g1_i1	sarcoplasmic/endoplasmic reticulum calcium ATPase 1 isoform X2	ATP2A1	16	21	-0.80	0.77	-1.72	**0.02**	**0.05**	**0.02**
115	TRINITY_DN38343_c2_g1_i2	myosin-1B-like isoform X4	MYO1B	19	42	-0.8	1.3	-2.1	0.11	0.32	0.17
116	TRINITY_DN25656_c0_g1_i1	myosin-3 isoform X1	MYH3	12	16	-0.89	0.97	-1.44	**0.03**	**0.01**	**0.05**
117	TRINITY_DN37664_c1_g1_i1	myosin-binding protein C, fast-type	MYBPC2	8	10	-0.95	1.01	-1.13	0.77	0.14	0.12
118	TRINITY_DN37754_c1_g1_i3	synaptophysin-like protein 2	SYPL2	1	2	-0.96	0.69	-2.25	**0.03**	**0.01**	0.13

119	TRINITY_DN38316_c1_g1_i3	nebulin isoform X9	NEB	14	15	-1.05	-0.16	-1.30	0.25	0.39	0.30
120	TRINITY_DN36517_c0_g2_i1	ATP-dependent 6-phosphofructokinase, muscle type	PFKM	10	14	-1.06	0.71	-1.32	**0.02**	**0.02**	**0.04**
121	TRINITY_DN38333_c1_g1_i1	glycogen debranching enzyme isoform X1	AGL	1	1	-1.09	0.74	-1.60	**0.04**	**0.03**	**0.02**
122	TRINITY_DN34542_c0_g1_i4	ovotransferrin-like isoform X1	TF	3	3	-1.12	2.41	-0.36	0.21	0.19	0.16
123	TRINITY_DN34011_c0_g1_i1	myosin heavy chain, skeletal muscle, adult	MYH1	6	11	-1.34	1.52	-2.63	0.37	0.23	0.16
124	TRINITY_DN27696_c0_g2_i4	alpha-2-macroglobulin-like	A2M	2	2	-1.61	2.11	-1.23	1.10	0.76	**0.04**
125	TRINITY_DN34296_c3_g4_i1	actin, cytoplasmic 2	ACTB	1	1	-1.85	-0.34	-1.16	**0.01**	**0.04**	**0.04**
126	TRINITY_DN28457_c0_g1_i3	titin isoform X50	TTN	2	2	-2.32	1.72	-1.89	**0.04**	**0.04**	0.09
127	TRINITY_DN33871_c5_g3_i3	feather keratin B-4-like	FKER	2	4	-3.19	0.85	-0.68	0.63	0.11	0.08
128	TRINITY_DN31256_c2_g1_i3	glyceraldehyde-3-phosphate dehydrogenase	GAPDH	5	10	-4.97	1.26	-1.78	0.62	0.39	1.10

Figure 2 – Supplementary Files

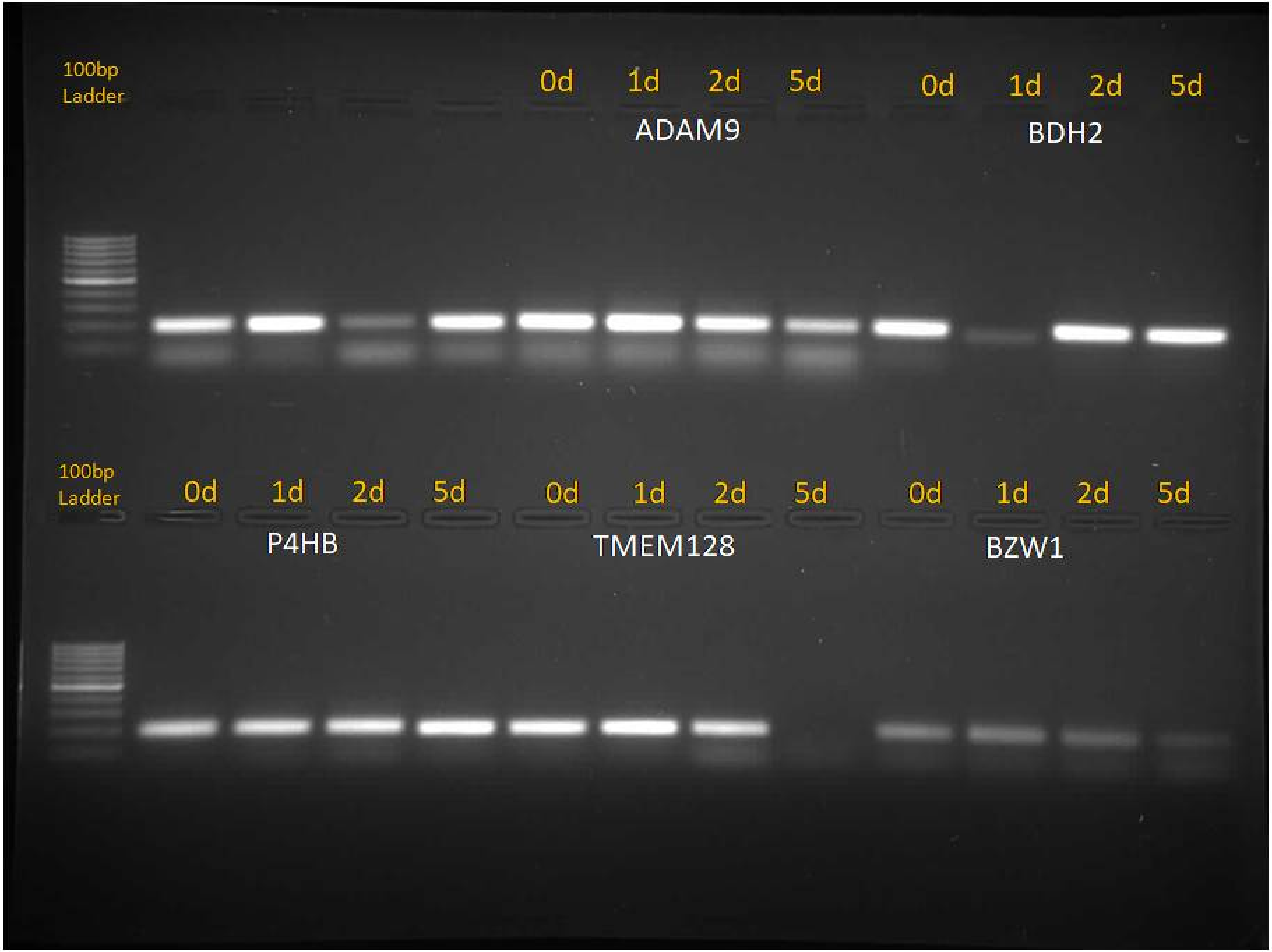
100bp Ladder
0d
1d
2d
5d
ADAM9
0d
1d
2d
5d
BDH2
100bp Ladder
0d
1d
2d
5d
P4HB
0d
1d
2d
5d
TMEM128
0d
1d
2d
5d
BZW1

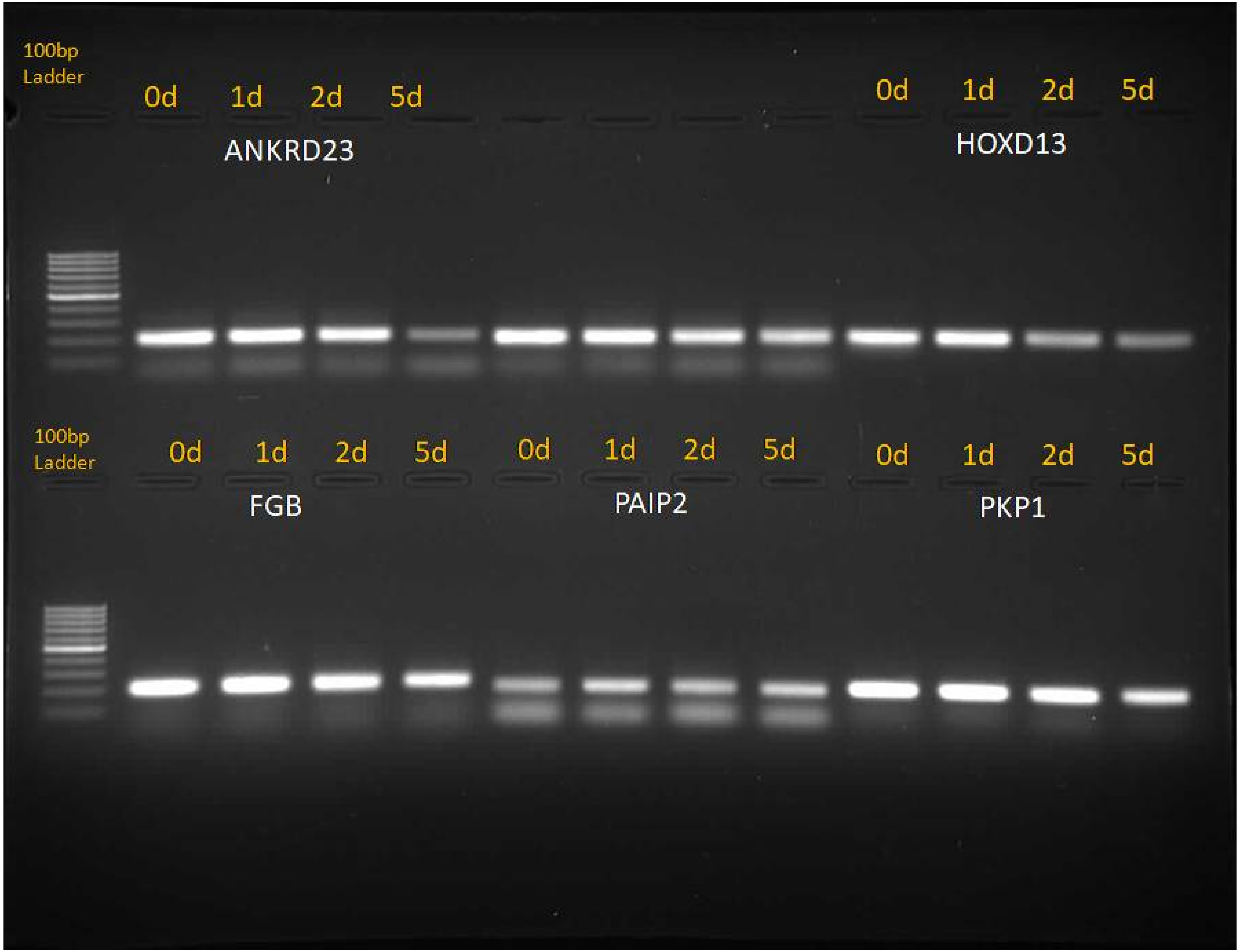
100bp Ladder
0d
1d
2d
5d
ANKRD23
0d
1d
2d
5d
HOXD13
100bp Ladder
0d
1d
2d
5d
FGB
0d
1d
2d
5d
PAIP2
0d
1d
2d
5d
PKP1

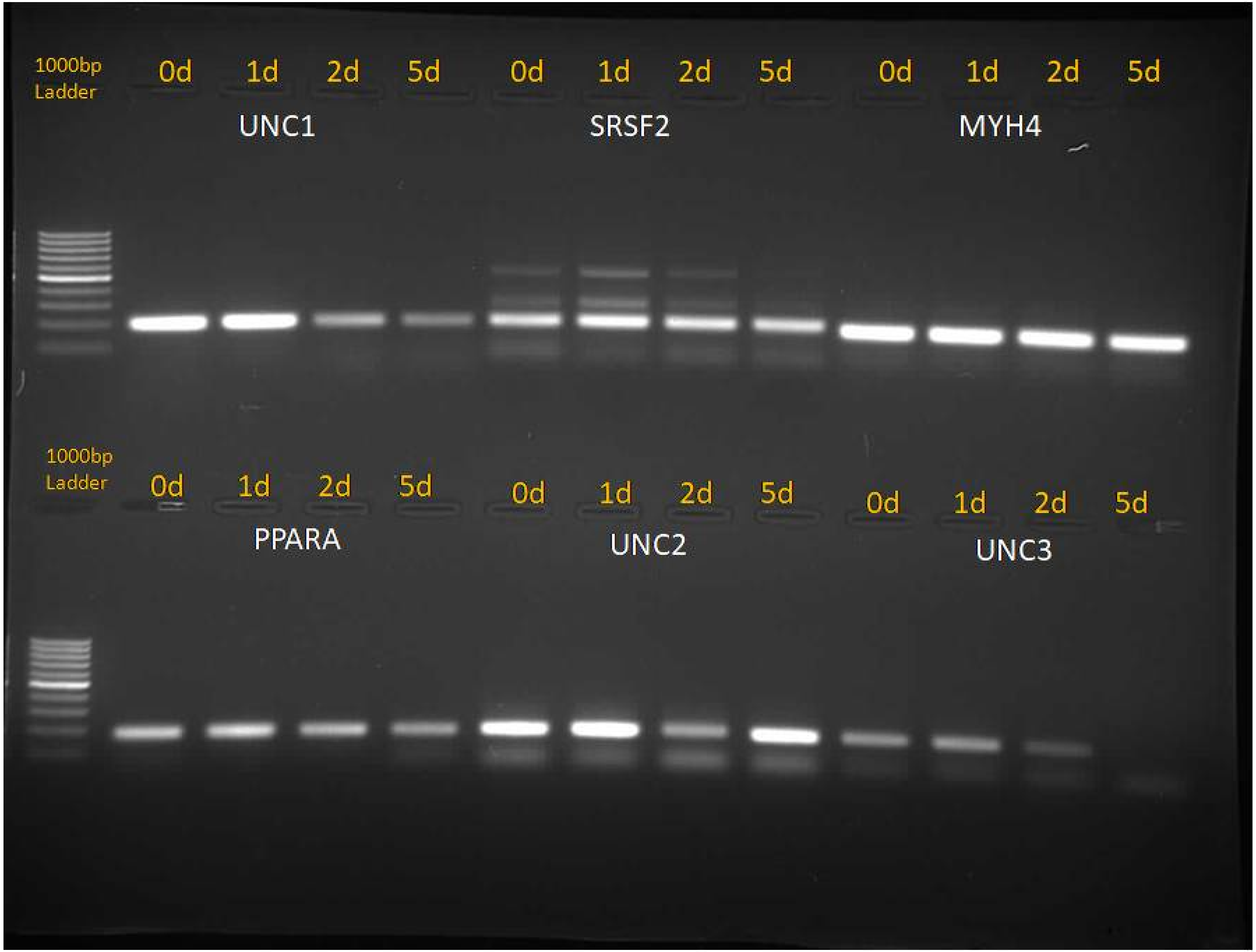
1000bp Ladder
0d
1d
2d
5d
UNC1
0d
1d
2d
5d
SRSF2
0d
1d
2d
5d
MYH4
1000bp Ladder
0d
1d
2d
5d
PPARA
0d
1d
2d
5d
UNC2
0d
1d
2d
5d
UNC3

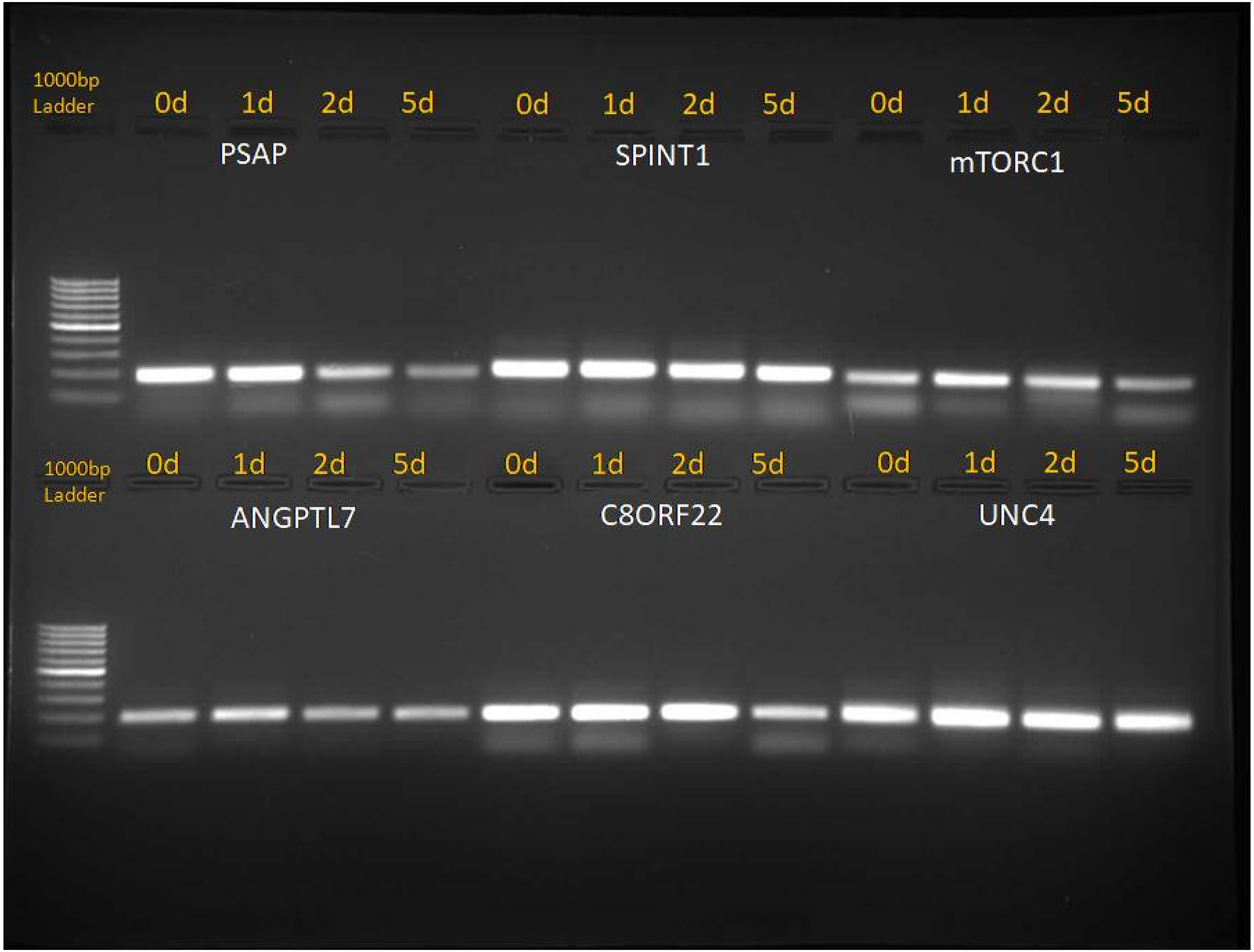

1000bp Ladder
0d
1d
2d
5d
PSAP
0d
1d
2d
5d
SPINT1
0d
1d
2d
5d
mTORC1
1000bp Ladder
0d
1d
2d
5d
ANGPTL7
0d
1d
2d
5d
C8ORF22
0d
1d
2d
5d
UNC4

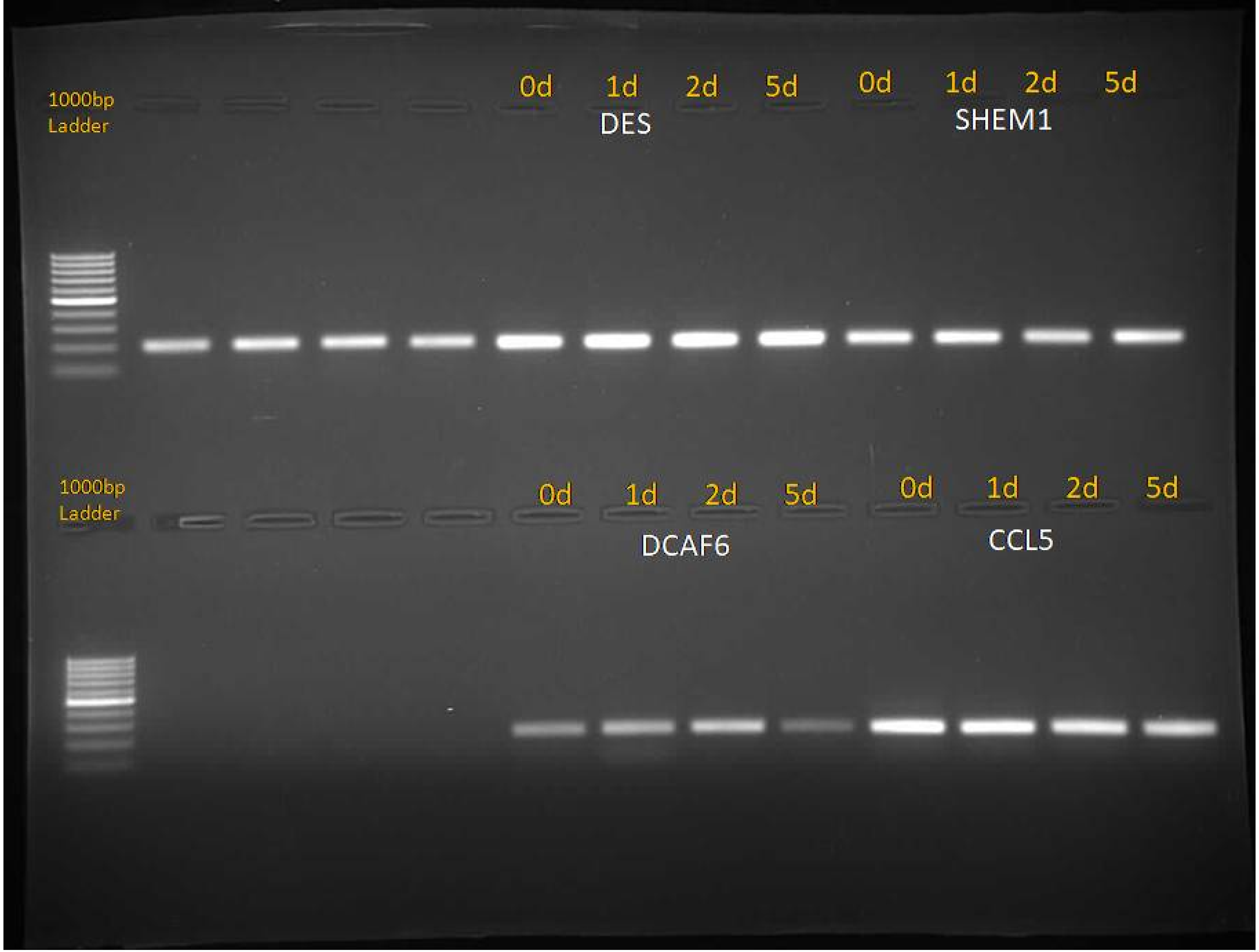
1000bp Ladder
0d
1d
2d
5d
DES
0d
1d
2d
5d
SHEM1
1000bp Ladder
0d
1d
2d
5d
DCAF6
0d
1d
2d
5d
CCL5

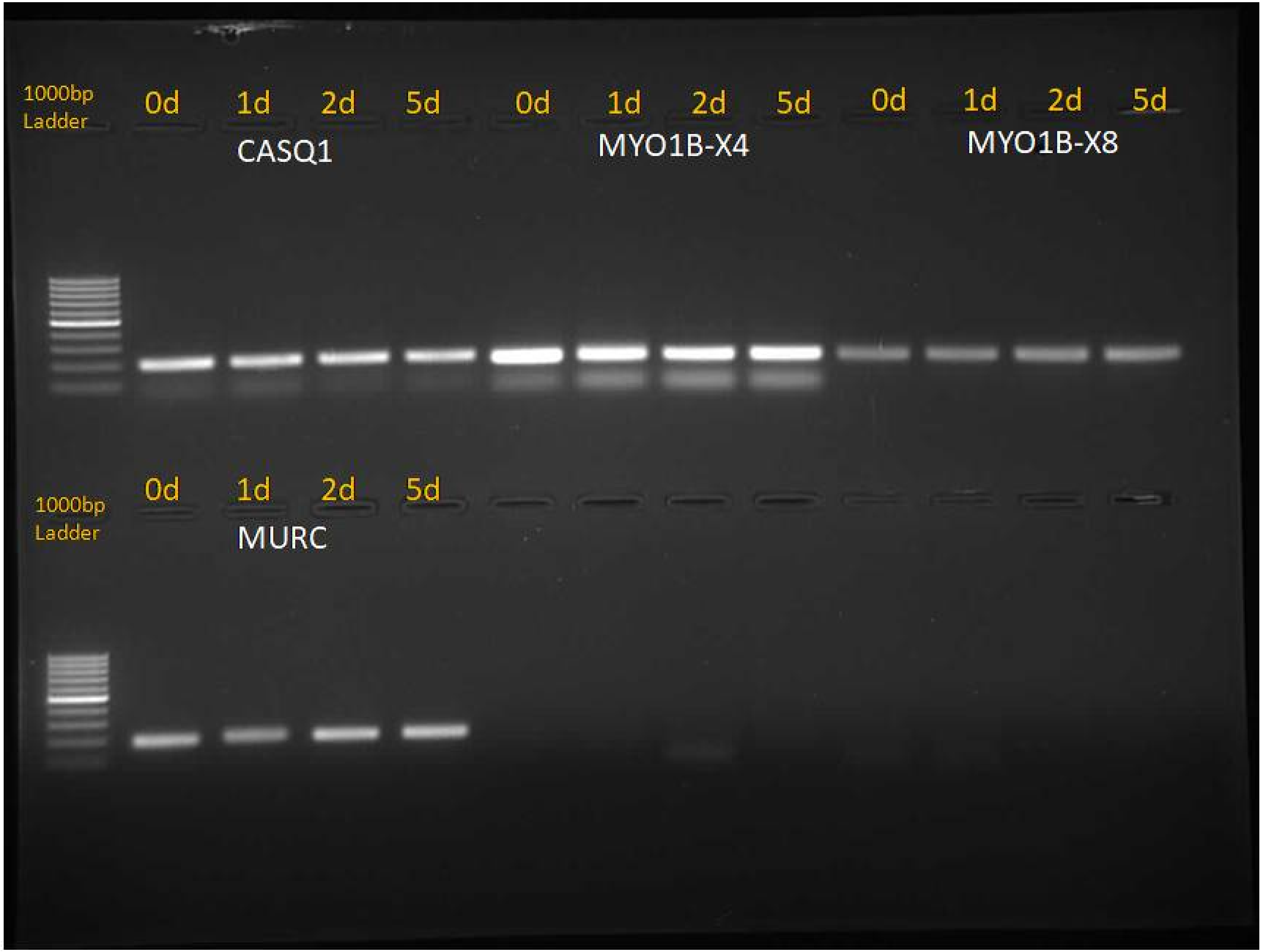
1000bp Ladder
0d
1d
2d
5d
CASQ1
0d
1d
2d
5d
MYO1B-X4
0d
1d
2d
5d
MYO1B-X8
1000bp Ladder
0d
1d
2d
5d
MURC

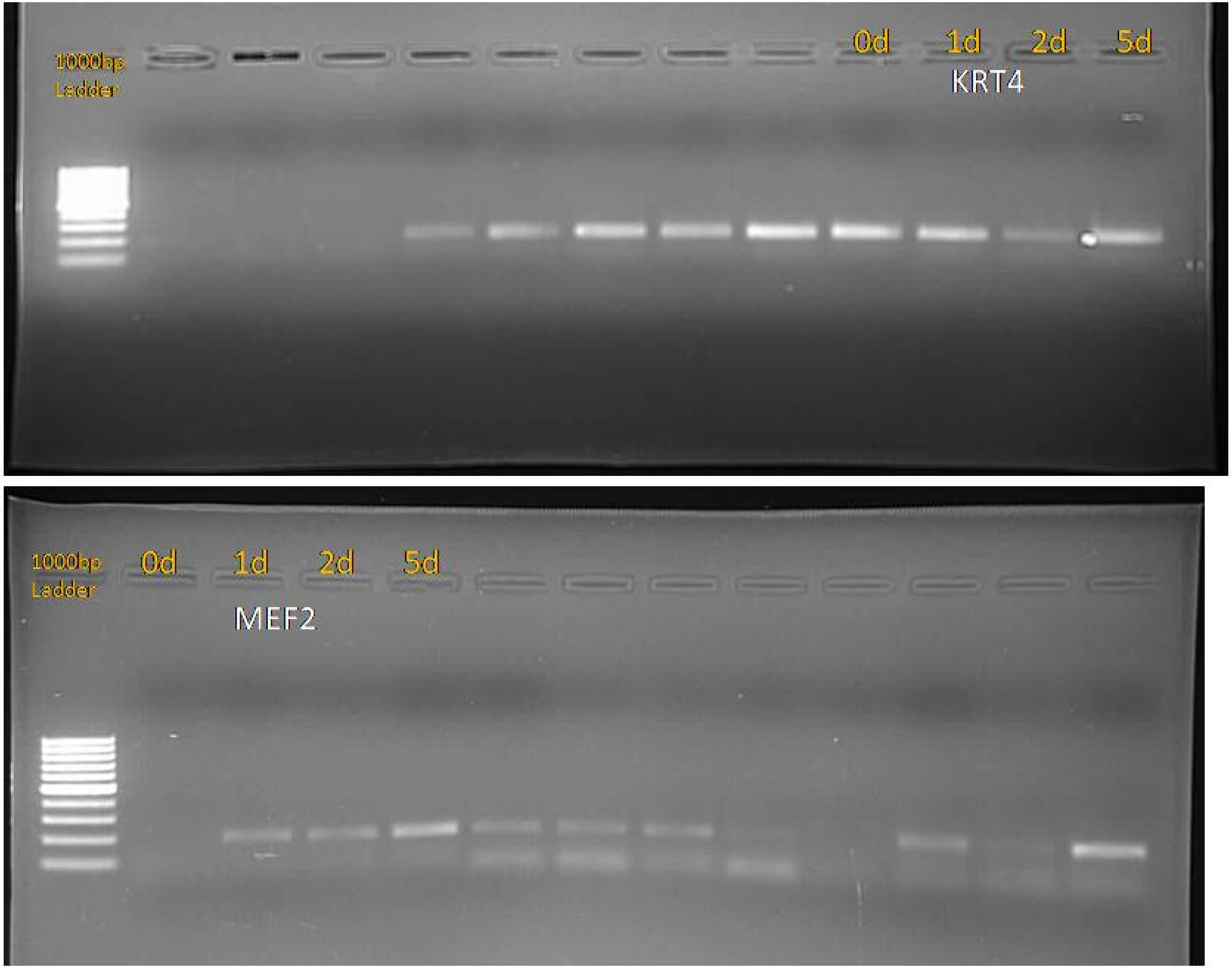
1000bp Ladder
0d
1d
2d
5d
KRT4
1000bp Ladder
0d
1d
2d
5d
MEF2

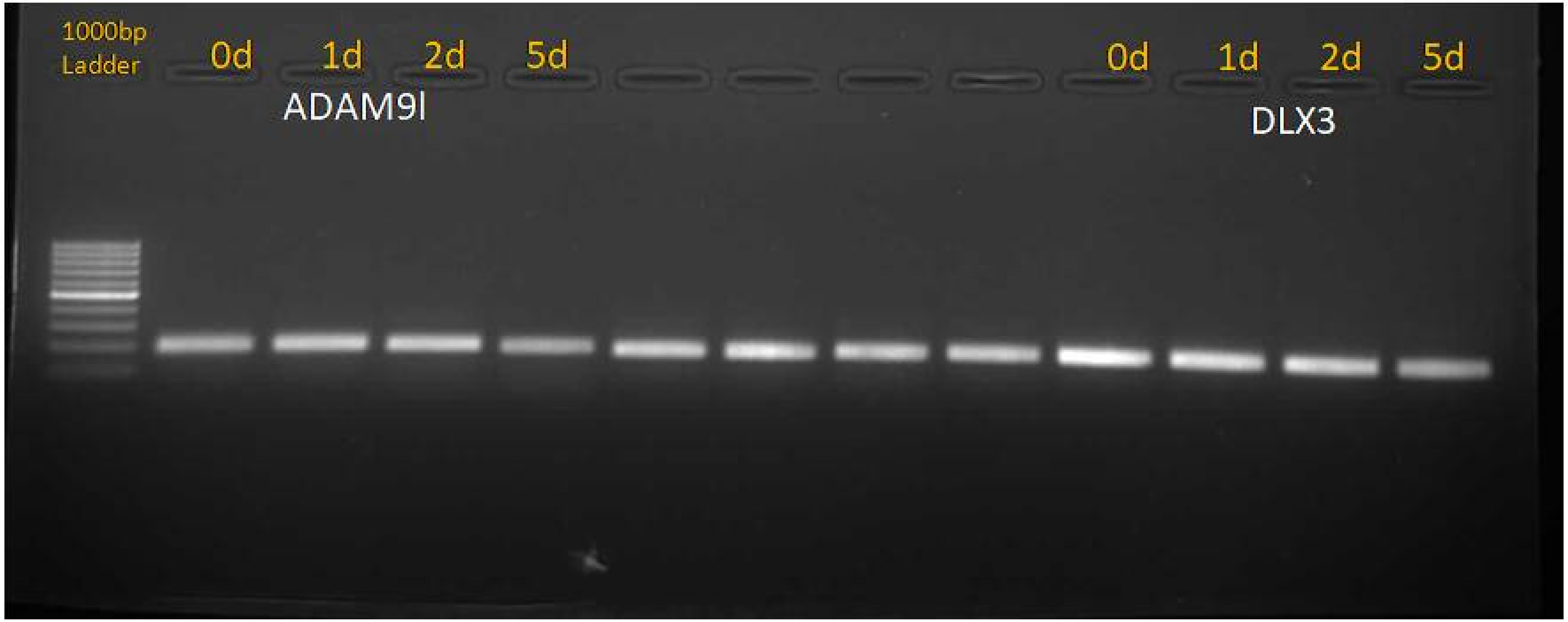
1000bp
Ladder
0d
1d
2d
5d
ADAM9l
0d
1d
2d
5d
DLX3

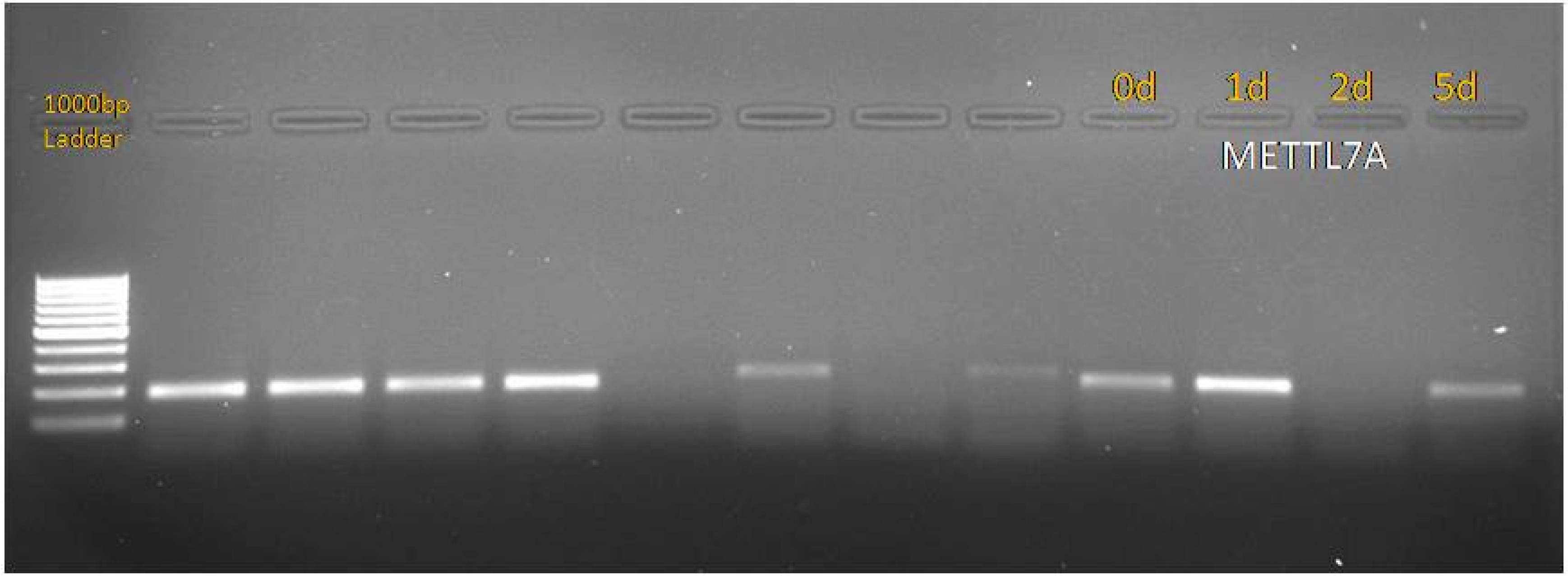
1000bp
Ladder
0d
1d
2d
5d
METTL7A

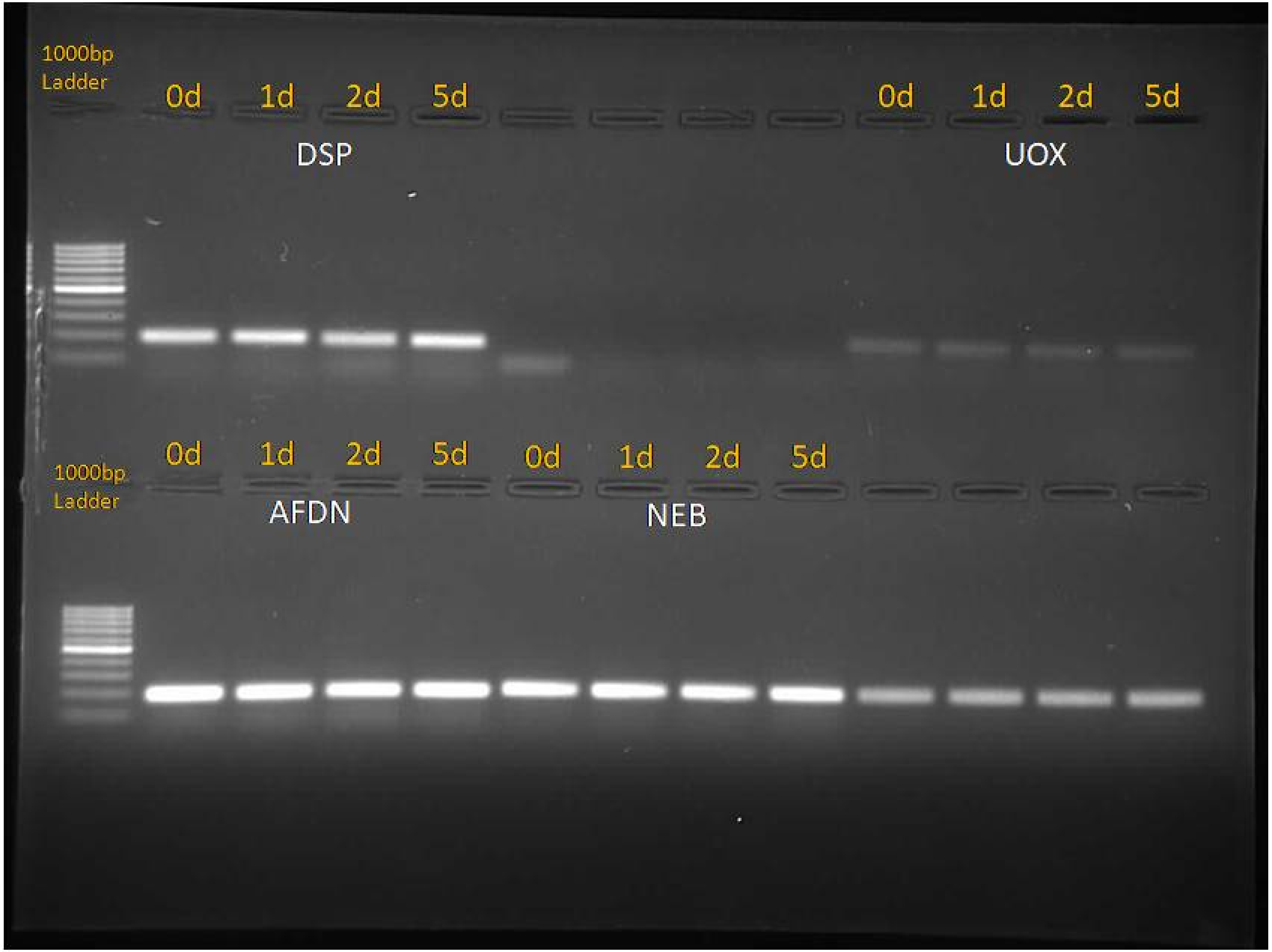
1000bp Ladder
0d
1d
2d
5d
DSP
0d
1d
2d
5d
UOX
1000bp Ladder
0d
1d
2d
5d
AFDN
0d
1d
2d
5d
NEB

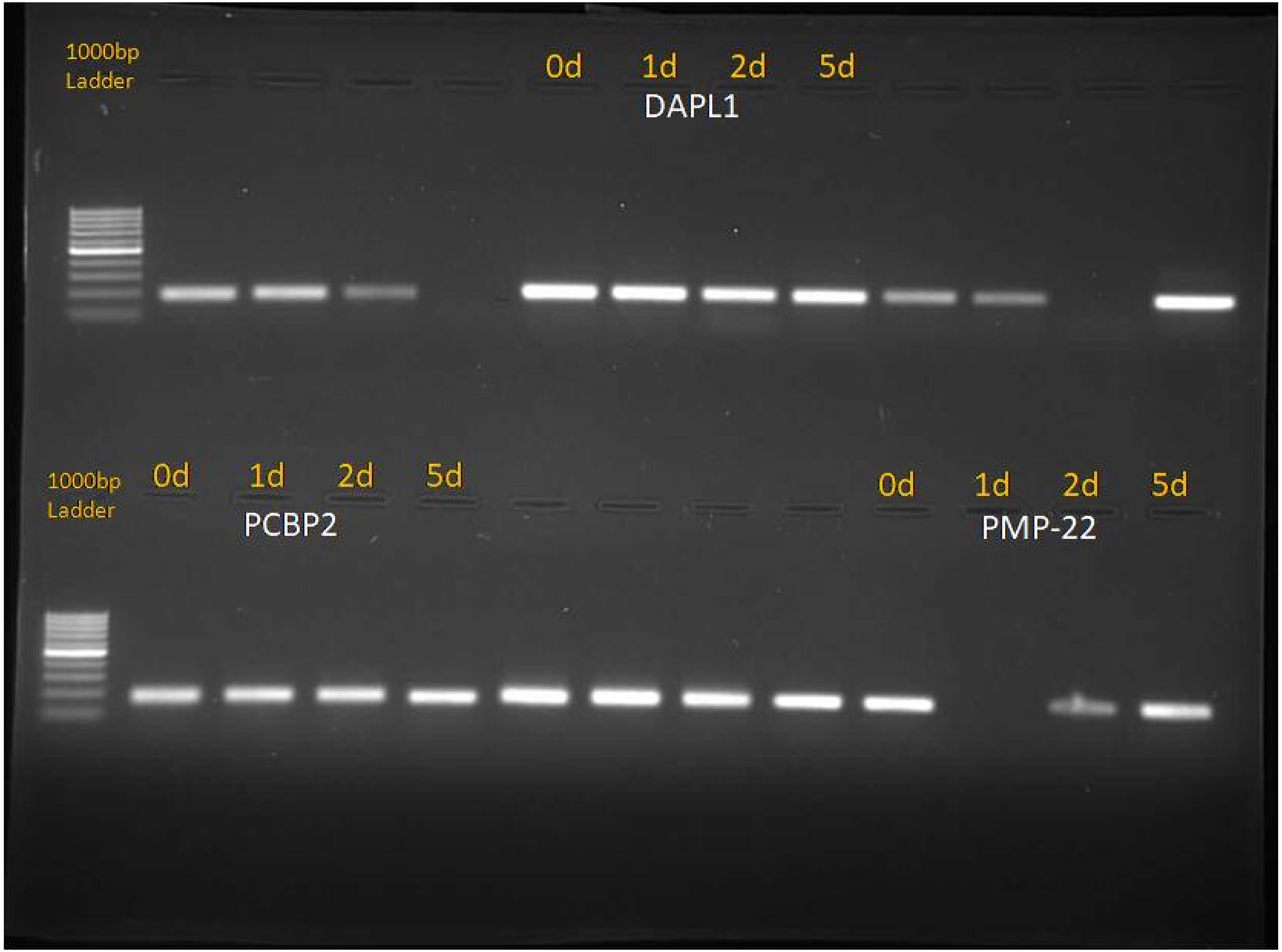
1000bp
Ladder
0d
1d
2d
5d
DAPL1
1000bp
Ladder
0d
1d
2d
5d
PCBP2
0d
1d
2d
5d
PMP-22

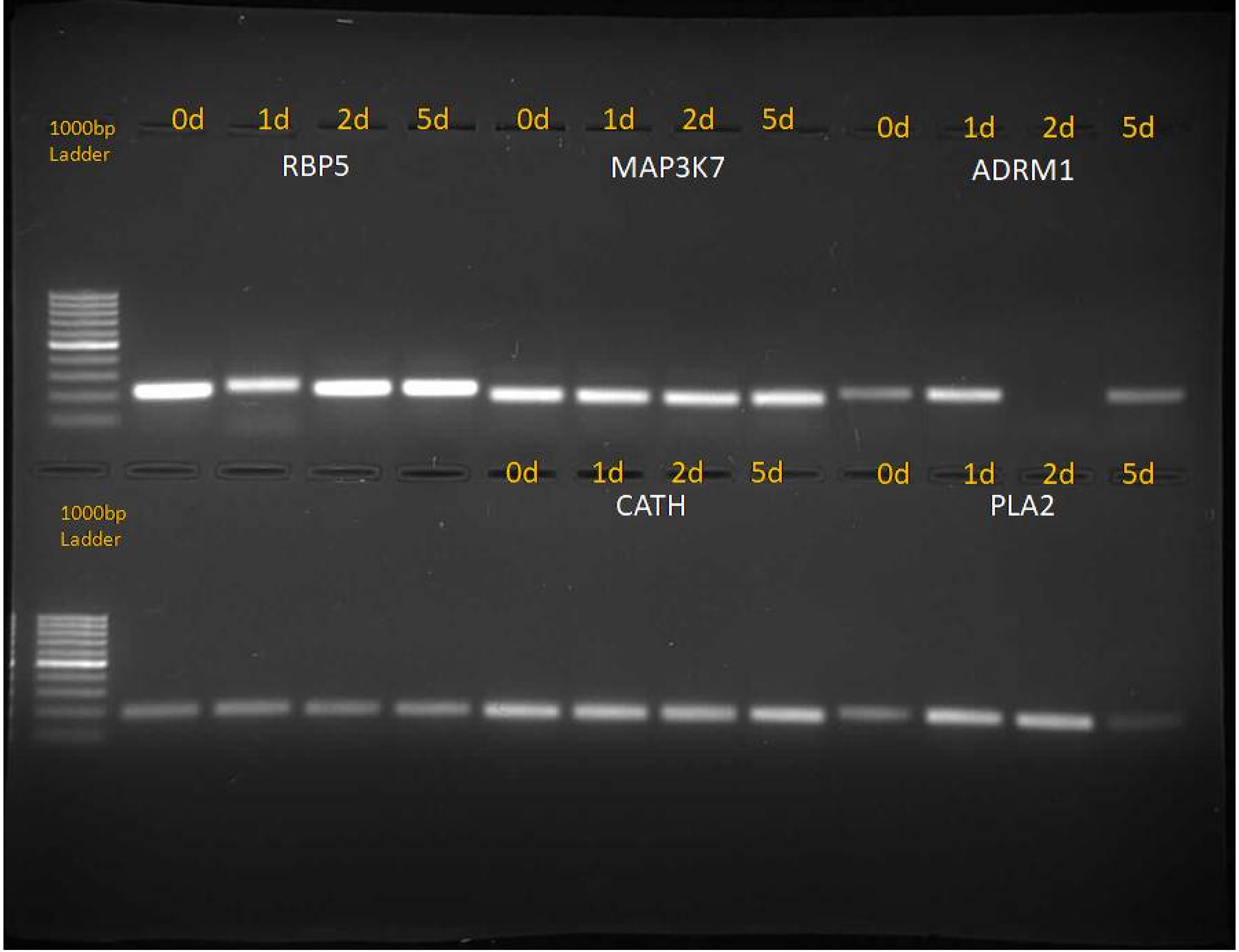
1000bp Ladder
0d
1d
2d
5d
RBP5
0d
1d
2d
5d
MAP3K7
0d
1d
2d
5d
ADRM1
1000bp Ladder
0d
1d
2d
5d
CATH
0d
1d
2d
5d
PLA2

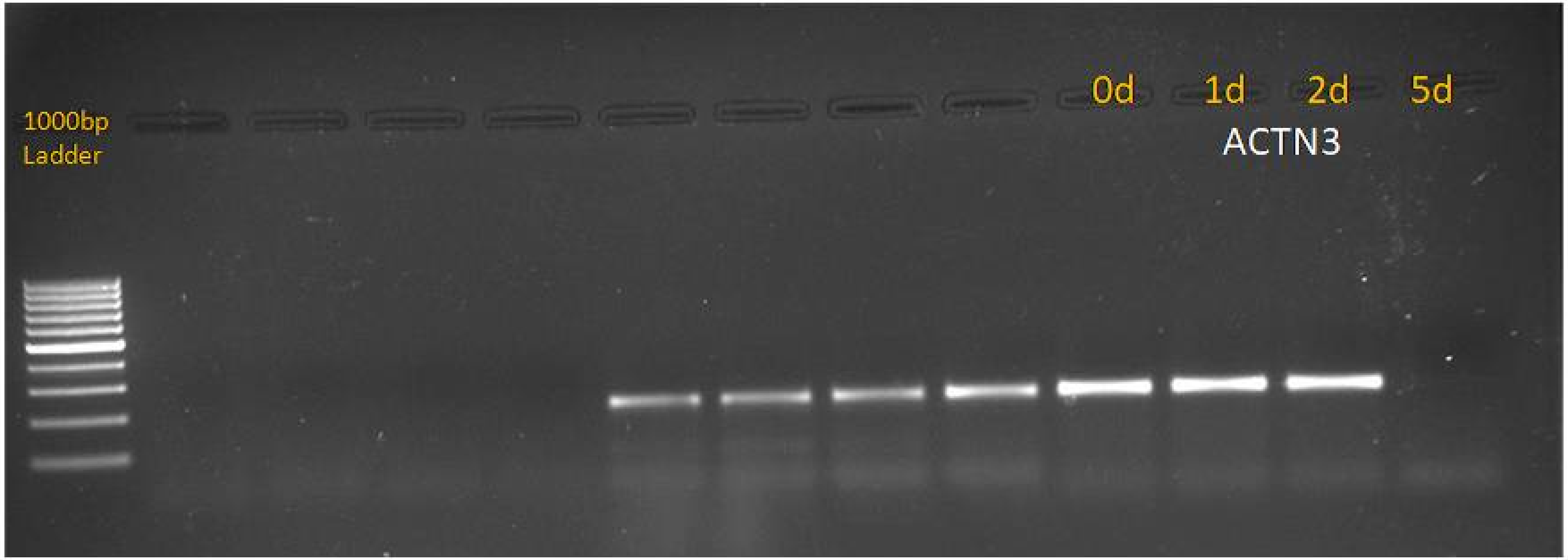
1000bp
Ladder
0d
1d
2d
5d
ACTN3

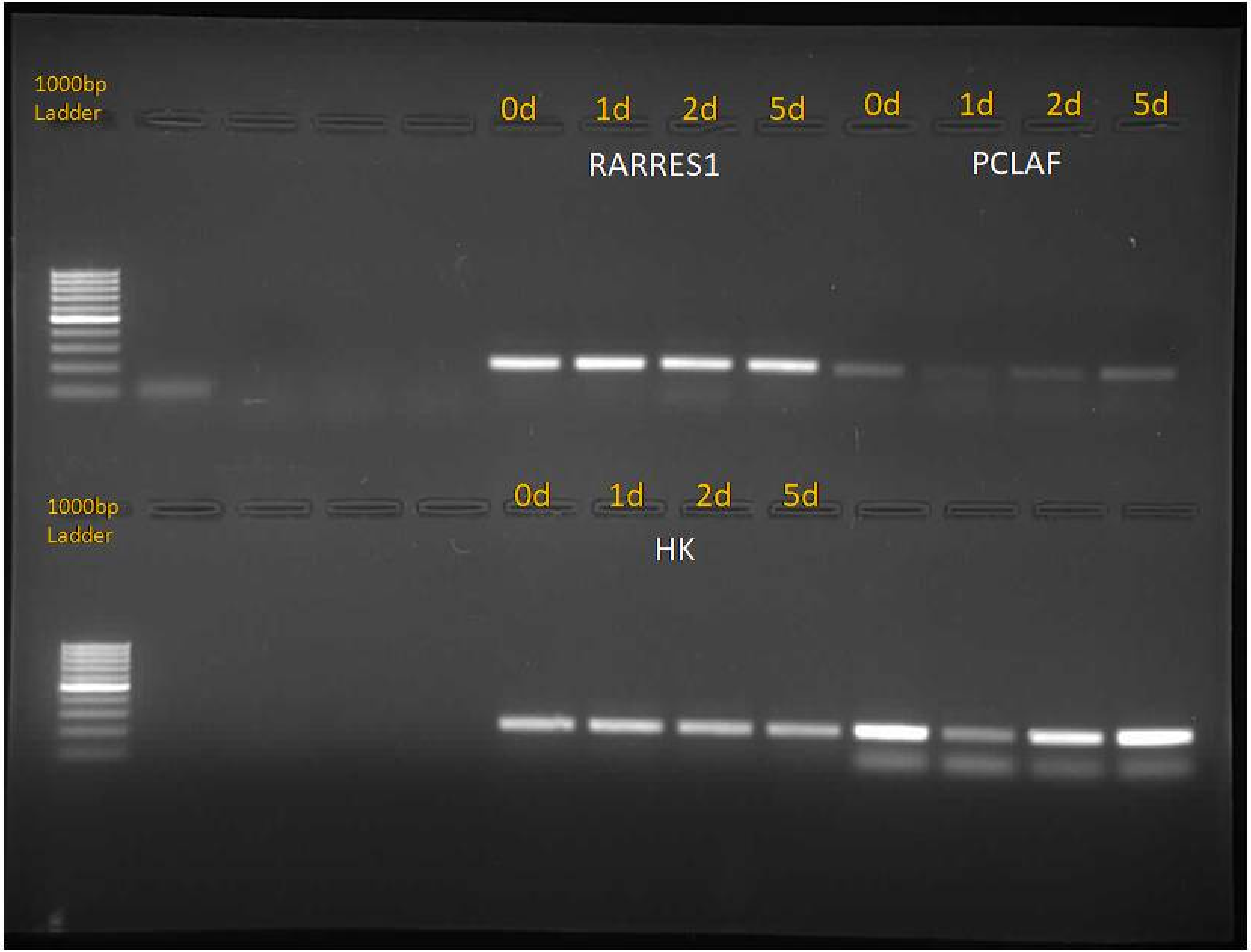

1000bp Ladder
0d
1d
2d
5d
RARRES1
0d
1d
2d
5d
PCLAF
1000bp Ladder
0d
1d
2d
5d
HK
